# Aroma volatile analyses and 2AP characterization at various developmental stages in Basmati and Non-Basmati scented rice (*Oryza sativa* L.) cultivars

**DOI:** 10.1186/s12284-016-0113-6

**Published:** 2016-08-05

**Authors:** Vidya R. Hinge, Hemant B. Patil, Altafhusain B. Nadaf

**Affiliations:** 1 Department of Botany, Savitribai Phule Pune University, Pune, 411007 India; 2 Department of Plant Biochemistry and Molecular Biology, Vilasrao Deshmukh College of Agricultural Biotechnology, Latur, VNMKV, Parbhani, 413512 India

**Keywords:** Rice, Odor active compounds (OAC’s), Volatile organic compounds (VOC’s), Head space solid phase microextraction (HS-SPME), Gas chromatography mass spectrometry (GCMS), Biomarker, Basmati

## Abstract

**Background:**

Rice plant growth is comprised of distinct phases, such as vegetative, reproductive, grain filling and maturity phases. In these phases synthesis and availability of primary and secondary metabolites including volatile organic compounds (VOC’s) is highly variable. In scented rice, aroma volatiles are synthesized in aerial plant parts and deposited in mature grains. There are more than 100 VOCs reported to be responsible for flavor in basmati rice. It will be interesting to keep track of aroma volatiles across the developmental stages in scented rice. Therefore, the aroma volatiles contributing in aroma with special reference to the major compound 2 acetyl-1-pyrroline (2AP) were screened at seven developmental stages in scented rice cultivars Basmati-370 and Ambemohar-157 along with non-scented rice cultivar IR-64 as a control following HS-SPME-GC-MS method. In addition, the expression levels of key genes and precursor levels involved in 2AP biosynthesis were studied.

**Results:**

The study indicated that volatilome of scented rice cultivars is more complex than non-scented rice cultivar. N-heterocyclic class was the major distinguishing class between scented from non-scented rice. A total of 14 compounds including, 2AP were detected specifically in scented rice cultivars. Maximum number of compounds were synthesized at seedling stage and decreased gradually at reproductive and maturity. The seedling stage is an active phase of development where maximum number green leaf volatiles were synthesized which are known to act as defense molecules for protection of young plant parts. Among the 14 odor active compounds (OACs), 10 OACs were accumulated at higher concentrations significantly in scented rice cultivars and contribute in the aroma. 2AP content was highest in mature grains followed by at booting stage. Gene expression analysis revealed that reduced expression of *betaine aldehyde dehydrogenase* 2 (*badh*2) and *glyceraldehyde-3-phosphate dehydrogenase* (*GAPDH*) and elevated level of *triose phosphate isomerase* (*TPI*) and Δ*1-Pyrolline-5-carboxylic acid synthetase* (*P5CS*) transcript enhances 2AP accumulation.

**Conclusions:**

Most diverse compounds were synthesized at seedling stage and OACs were accumulated more at flowering followed by seedling stage. Distinct accumulation pattern exists for 2AP and other aroma volatiles at various developmental stages. The study revealed the mechanism of 2AP accumulation such that 2AP in mature grains might be transported from leaves and stem sheath and accumulation takes place in grains.

**Electronic supplementary material:**

The online version of this article (doi:10.1186/s12284-016-0113-6) contains supplementary material, which is available to authorized users.

## Background

Rice (*Oryza sativa* L.) is an annual crop cultivated for grains as a rich source of carbohydrates minerals and vitamins. Based on developmental pattern, rice plant growth is divided into vegetative (germination to panicle initiation), reproductive (panicle initiation to heading), grain filling or ripening and maturity phases. At seedling stage the plant has clearly defined shoot and root parts. Further tillers are formed on main shoot, a gradual increase in plant height and leaf emergence at regular intervals takes place during vegetative development. In reproductive stage plant undergo culm elongation, a decline in tiller number, booting, emergence of the flag leaf, heading or panicle protrude out from sheath and flowering. Grain filling and ripening or maturation stage is characterized by grain growth (Yoshida and Nagota 2011). The developmental phases have direct and indirect influences on the yield and quality characteristics of rice grains. The reproductive growth stage is most sensitive to biotic and abiotic stresses (Fageria 2007). As the plant undergo different developmental phases, simultaneous level of endogenous hormones, primary and secondary metabolites including volatile organic compounds (VOCs) are altered. Photosynthates produced in leaves are transported primarily in the form of sucrose to meristem and developing organs such as flowers. During this phase of development, maximum amino acids and proteins are synthesized in plants and transported to developing organs where they are utilized for flower and embryo development. At grain filling or ripening, grain size and weight increases since starch and sugars are translocated from the culms and leaf sheaths to developing grains. The carbohydrate is stored in the form of starch in grain endosperm. Initially when florets on the main stem show milky accumulation, starch is white and milky in consistency. It loses moisture and changes into bread dough or firmer during dough grain stages. At maturity physiological process of grain filling cease. And when the moisture content of the grain on the main stem is 25 to 30 % the plant reaches to physiological maturity (Paul and Foyer 2001).

A relatively large group of plant natural products consists of VOCs, lipophilic liquids with low molecular weight and high vapor pressure are synthesized during growth developmental stages. Physical properties of these compounds allow them to freely cross cellular membranes and be released into the surrounding environment (Pichersky et al. 2006). Over the years > 1700 VOCs have been identified from 90 different plant families belonging to both angio- and gymnosperms (Knudsen et al. 2006). Biosynthesis of VOCs depends on the availability of carbon, nitrogen and sulfur as well as energy provided by primary metabolism. Biosynthesis of the wide array of different VOCs branches off from only a few primary metabolic pathways. Based on their biosynthetic origin, all VOCs are divided into several classes, including terpenoids, phenylpropanoids (benzenoids), fatty acid derivatives and amino acid derivatives etc. (Dudareva et al. 2013). There are more than 100 volatile compounds were reported responsible for basmati flavor representing 13 hydrocarbons, 14 acids, 13 alcohols, 16 aldehydes, 14 ketones, 8 esters, 5 phenols etc. (Hussain et al. 1987). 2-Acetyl-1-pyrroline (2AP), a principal aroma compound has been detected in all aerial plant parts of scented rice (Yoshihashi et al. 2002a, b; Maraval et al. 2010). Recently, in addition to 2AP, several other major volatiles contributing in the aroma of scented rice cultivars have been identified viz., hexanal, nonanal, octanal, (E)-2-nonenal, (E,E)-2,4-nonadienal, heptanal, pentanal, (E)-2-octenal, 4-vinylphenol, 4-vinylguaicol,1-octen-3-ol, decanal, guaicol, indole and vanillin (Mathure et al. 2011; Mathure et al. 2014). It is interesting to keep track of aroma volatiles synthesized and translocated across various developmental stages in scented rice. Such studies have not yet been reported in any scented rice cultivars. Therefore, in present study 2AP, other aroma volatiles and 2AP precursors (proline and methylglyoxal) were assessed at seven different growth stages in two scented rice cultivars Basmati-370 (BA-370) and Ambemohar-157 (AM-157) and compared with non-scented rice cultivar IR-64. In addition, the expression analysis of genes involved in the biosynthesis of 2AP [*betaine aldehyde dehydrogenase* 2 (*badh*2), Δ*1-Pyrolline-5-carboxylic acid synthetase (P5CS)*, *triose phosphate isomerase(TPI)* and *glyceraldehyde-3-phosphate dehydrogenase (GAPDH)*] have been studied.

## Results and discussion

### Qualitative analysis of aroma volatiles

The volatile compounds identified in three rice cultivars at seven developmental stages (S1 to S7) are depicted in Table 1. Total 88 volatile compounds were identified collectively in three rice cultivars which belongs to 13 chemical classes viz*.* alkane (7), alkene (6), ketone (12), aromatic hydrocarbon (4), terpenes (11), alcohols (13), aliphatic aldehydes (16), aromatic aldehydes (3), N-heterocyclic (3), ester (7), phenol containing compounds (4), carboxylic acid (1) and furan (1). In scented rice cultivars more number of volatile compounds (72–51) were detected throughout the developmental stages than the non-scented one (58–39). In either one or all stages, 14 compounds were detected specifically in scented rice cultivars. In this 14 compounds eight compounds [2AP (nutty’ or ‘popcorn-like), 2-acetyl-1H-pyrrole (musty and nutty), β-ionone (raspberry, floral), (E,Z)-2,6-nonadienal (green, metallic), p-xylene (sweetish), methyl 2-aminobenzoate (sweet, grape fruity) having characteristic odor and azulene and acetic acid, 1,7,7-trimethyl-bicyclo(2,2,1)hept-2-yl, ester without any contribution in odor] were commonly detected in both scented rice cultivars. Among the scented cultivars, 5 compounds [4-methyldecane (pungent, acrid odor), 4-cyclopentylidene-2-butanone (woody and fruity), toluene (sweet, pungent, benzene), indole (floral, sweet, burnt) and allylcyclohexane (fruity, pineapple)] were detected specifically in AM-157 and camphene (woody, camphoreous) was detected only in BA-370. In present study this five compounds in AM-157 and single compound in BA-370 was observed cultivar specific. In comparative volatile analysis of aromatic and non-aromatic rice cultivars no difference was recorded in presence of volatile compounds but only in the quantity (Widjaja et al. 1996). Other studies showed that in comparative volatiles analysis of mature grains of scented and non-scented rice not a single compound or set of compounds defined a particular aromatic rice cultivar but only the concentration (Jezussek et al. 2002; Zeng et al. 2009). Recently, Bryant and Mcclung (2011) reported presence of 15 compounds in addition to 2AP in mature grains of seven aromatic cultivars over non-aromatic rice cultivars. Similar results were recorded here.

**Table 1:** List of volatile compounds identified at various developmental stages in 3 rice cultivars

Sr.No	Compound	Odor description	Retention Index (RI)		AM-157	BA-370	IR-64
			Exp	Ref	Stage of occurrence		
1. Alkane							
1	Nonane	Gasoline-like	901	900	S4, S5, S6	S4 to S7	S5, S6
2	4-Methyldecane^b^	Pungent,	1022	1023	S7	ND	ND
3	Dodecane	Gasoline like	1196	1200	S1, S4 to S7	S1, S4 to S7	S1, S4 to S7
4	Tetradecane^a^	Gasoline-like	1390	1400	S1 to S7	S1 to S7	S1 to S7
5	Pentadecane^a^	Mild odor	1501	1500	S1 to S7	S1 to S7	S1 to S7
6	Heptadecane^a^	NA	1698	1700	S1 to S7	S1 to S7	S1 to S7
7	Nonadecane^a^	Sweet, rosy	1898	1900	S1 to S7	S1 to S7	S1 to S7
2. Alkene							
1	Allylcyclohexane^b^	NA	965	969	S2, S3	S1 to S3	ND
2	(E)-5-Methyl-4-decene	NA	1094	1100	S1 to S3, S5 to S7		S2,S5, S6
3	(Z)-3-Undecene	Hydrocarbon	1116	1123	S2,S3,S4 - S6	S1- S3, S7	S1, S3
4	(Z)-3-Dodecene	Pleasant odor	1194	1195	S1,S2, S4 - S6	S1,S2,S4-S7	S1, S4-S7
5	7-Tetradecene	NA	1362	1367	S1, S2, S7	S1 to S3	S1 to S3
6	1-Tetradecene^a^	Mild pleasant	1392	1385	S1 to S7	S1 to S7	S1 to S7
3. Ketone							
1	2-Heptanone^a^	Fruit,spicy	896	889	S1-S7	S1-S7	S1-S7
2	6-Methyl-2-heptanone^a^	Camphoreous	956	957	S1-S4, S7	S1-S4, S7	S1-S4, S7
3	6-Methyl-5-hepten-2-one^a^	Herby, green	987	988	S1-S7	S1-S7	S1-S7
4	(E)-3-Octen-2-one^a^	Citrus, floral	1040	1036	S1,S2, S6,S7	S1,S2,S4,S6,S7	S1,S2,S6,S7
5	2,2,6-Trimethylcyclohexanone	Citrus, hone	1043	1047	S1-S4	S1-S4	S1-S4
6	2-Nonanone	Green, herbal	1094	1093	S1, S2, S5	S1, S2, S7	S1, S2
7	(E)-5-Ethyl-6-methyl-3-hepten-2-one	Citrus type	1147	1144	S1, S6, S7	S1, S3-S7	S6, S7
8	4-Cyclopentylidene-2-butanone^b^	Woody, fruity	1151	1158	S7	ND	ND
9	2,6,6-Trimethyl-2-cyclohexene-1,4-dione	Musty, citrus	1156	1152	S1-S6	S1-S4, S6-S7	S1-S7
10	2-Undecanone	Floral, pineapple	1301	1302	S5	S4	S4
11	6,10-Dimethyl-2-undecanone	NA	1404	1398	S3, S5-S7	S1-S3, S5-S7	S3, S5-S7
12	β-lonone^b^	Raspberry, floral	1495	1493	S1, S2	S2, S7	ND
4. Aromatic Hydrocarbon							
1	p-Xylene^b^	Strong, Sweetish	897	907	S1	S1	ND
2	Toluene^b^	Sweet, Pungent,	995	1005	S7	ND	ND
3	1-Isopropyl-2-methylbenzene	Woody, Smoky	1025	1025	S1-S3	S1-S3	S2
4	1-Isopropyl-4-methylbenzene	Woody, Smoky	1027	1023	S1-S3, S5	S1-3, S5	S2
5. Terpenes							
1	Pinene	Herbal type odor	935	930	S2	S1-S3, S5	S1, S2
2	Camphene^b^	Woody camphoreous	950	958	ND	S2, S5	ND
3	3-Carene^a^	Sweet & pungent	1014	1015	S1-S6	S1-S6	S1-S6
4	L-Limonene	Lemon	1038	1040	S1	S5	S5
5	Azulene^a^	NA	1324	1323	S7	S3	ND
6	β-Elemene	Fruity, Dry	1405	1403	S1-S3	S1-S3	S1, S2
7	Isolongifolene	Fresh woody	1420	1416	S1-S5	S1-S5	S1-S5
8	Longifolene	vegetal/flowery	1437	1432	S1-S6	S1-S7	S1-S6
9	β-Caryophyllene	Spicy type odor	1443	1444	S1-S6	S1-S7	S1-S6
10	Aromadendrene ^a^	Woody type odor	1447	1439	S1	ND	S1
11	Valencen	Sweet, citrus	1472	1477	S1-S6	S1-S6	S5-S6
6. Alcohol							
1	1-Pentanol^a^	Moderately strong	803	792	S1-S7	S1-S7	S1-S7
2	(Z)-3-Hexen-1-ol^a^	Green type odor	880	872	S1-S6	S1-S7	S1-S6
3	1-Hexanol^a^	Green	866	860	S1-S7	S1-S7	S1-S7
4	1-Octen-3-ol^a^	Straw, mushroom	972	969	S1-S7	S1-S7	S1-S7
5	1-Hexanol, 2-ethyl-	Heavy, earthy	1030	1033	S4-S7	S4, S5, S7	S3-S5
6	1-Octanol^a^	Fatty, metallic	1073	1073	S1-S7	S1-S7	S1-S7
7	Linalool^a^	Sweet, floral,	1102	1100	S1-S3,S5,S7	S1, S2, S4, S7	S1-S2, S4-S7
8	3, 4-Dimethylcyclohexanol	NA	1123	1126	S2-S6	S1-S7	S1-S7
9	2 Nonen-1-ol^a^	Green type	1134	1135	S1-S3,S5, S7	S1-S3, S5-S7	S1,S2, S4-S7
10	Carveol	Spicy type	1161	1188	S1,S3, S6,S7	S1, S2, S6, S7	S1-S3, S5-S7
11	3,7-Dimethyl-1-octanol	Sweet, rosy	1204	1196	S2, S7	ND	S5
12	2-Hexyl-1-octanol^a^	Waxy	1600	1591	S1-S7	S2, S3, S5-S7	S1-S3, S5-S7
13	2-Hexadecanol	NA	1798	1774	S1-S7,	S2-S3, S5-S7	S1-S3,S5, S6
7. Aliphatic Aldehyde							
1	Pentanal^a^	Strong, Acrid	710	707	S1-S7	S1-S7	S1-S7
2	Hexanal^a^	Green	816	820	S1-S7	S1-S7	S1-S7
3	(E)-2-Hexenal	Sweet, fruity, fresh	869	860	S2, S3	S1-S3, S5, S6	S2
4	Heptanal^a^	Grass, fresh	904	905	S1-S7	S1-S7	S1-S7
5	(Z)-2-Heptenal	Fatty green	961	960	S2-S7	S1-S7	S3-S7
6	Octanal^a^	Citrusy	1007	1005	S1-S7	S1-S7	S1-S7
7	(E,E)-2 4-Octadienal^a^	musty, cooked starch	1017	1021	S1-S7	S1-S7	S1-S6
8	(E)-2-Octenal^a^	nutty, cooked flour	1063	1068	S1-S7	S1-S3, S5-S7	
9	Nonanal^a^	Grassy, citrus, floral	1108	1104	S1-S7	S1-S7	S1-S7
10	(E,Z)-2,6-Nonadienal^b^	Green, metallic	1155	1153	S1, S5, S7	S2	ND
11	(E)-2-Nonenal	Metallic	1165	1162	S1-S7	S1-S7	S1-S7
12	Decanal^a^	Fatty, citrusy	1210	1204	S1-S7	S1-S7	S1-S7
13	(E,E)-2,4-Nonadienal	Fatty, metallic	1219	1217	S1-S3, S7	S1-S3	S1, S2
14	β-Cyclocitral	Minty type odor	1234	1237	S1-S7	S1-S7	S1-S7
15	(2,6,6-Trimethyl-1-cyclohexen-1- yl)acetaldehyde	NA	1269	1261	S1-S6	S1-S4, S6	S1-S6
16	(E,E)-2,4-Decadienal	Fatty, metallic, citrus	1325	1318	S1-S5, S7	S1-S7	S1-S4
8. Aromatic aldehyde							
1	Benzaldehyde^a^	Nutty,sweet	970	965	S1-S7	S1-S7	S1-S7
2	Phenylacetaldehyde^a^	Herbal, floral	1052	1048	S1-S7	S1-S7	S1-S7
3	Vanillin^a^	Vanilla-like	1415	1403	S1-S7	S1-S7	S1-S7
9. N-Heterocyclic							
1	2-Acetyl-1-pyrroline^ab^	popcorn-like	925	930	S1-S7	S1-S7	ND
2	1-(1H-Pyrrol-2-yl) ethanone^b^	Musty and nutty	1043	1035	S1, S3-S7	S1-S7	ND
3	1H-Indole^ab^	Floral, sweet, burnt	1306	1304	S1-S3	ND	ND
10. Ester							
1	Ethyl hexanoate	Fruity,	989	984	S1	S1	S1
2	Ethyl heptanoate	Berry like odor	1088	1083	S1	S1	S1
3	Ethyl octanoate	Waxy type	1193	1183	S1, S2	S1, S2	S1, S2
4	Methylsalicylate	Minty type odor	1201	1206	S1-S6	S1-S7	S1-S7
5	Acetic acid, 1,7,7-trimethyl-bicyclo(2,2,1)hept-2-yl, ester^b^	NA	1285	1277	S1	S2, S5	ND
6	Methyl 2-aminobenzoate^b^	sweet, grape fruity	1365	1372	S2, S4	S1, S2, S4	ND
7	Ethyl laurate^b^	Waxy type	1586	1581	S1, S2, S3	S1,S3	S1
11. Phenol-containing							
1	Phenol^a^	Sweet and tarry	981	980	S1, S2, S3	S1-S3, S5	S1-S3
2	2-Methoxyphenol^a^	Smoky	1087	1090	S1-S7	S1-S7	S1-S7
3	2-Phenoxyethanol	Pleasant odor	1224	1226	S2-S6	S1-S6	S3-S6
4	2-Methoxy-4-vinylphenol^a^	Spicy,fruity	1320	1313	S1-S7	S1-S7	S1-S6
12. Carboxylic acid							
1	Benzoic acid^a^	Pleasant odor	1179	1174	S1-S4	S1-S4	S1-S3
13. Furans							
1	2-Pentylfuran^a^	Floral, fruit, nutty	992	996	S1-S7	S1-S7	S1-S7
Total 88 compounds							

^a^Compound confirmed using standard

^b^Compound detected only in scented rice cultivars, *Expt* experimental, *Ref* reference, *NA* not avaliable., *ND* not detected, S1 seedling, S2 tillering, S3 booting, S4 flowering, S5 milky grains, S6 dough grains, S7 mature grains., *AM-157* ambemohar-157., *BA-370* Basmati-370

The qualitative analyses of volatile compounds at respective developmental stages are summarized as follows.

### Vegetative stages (S1: seedling and S2: tillering)

**Fig. 1:**
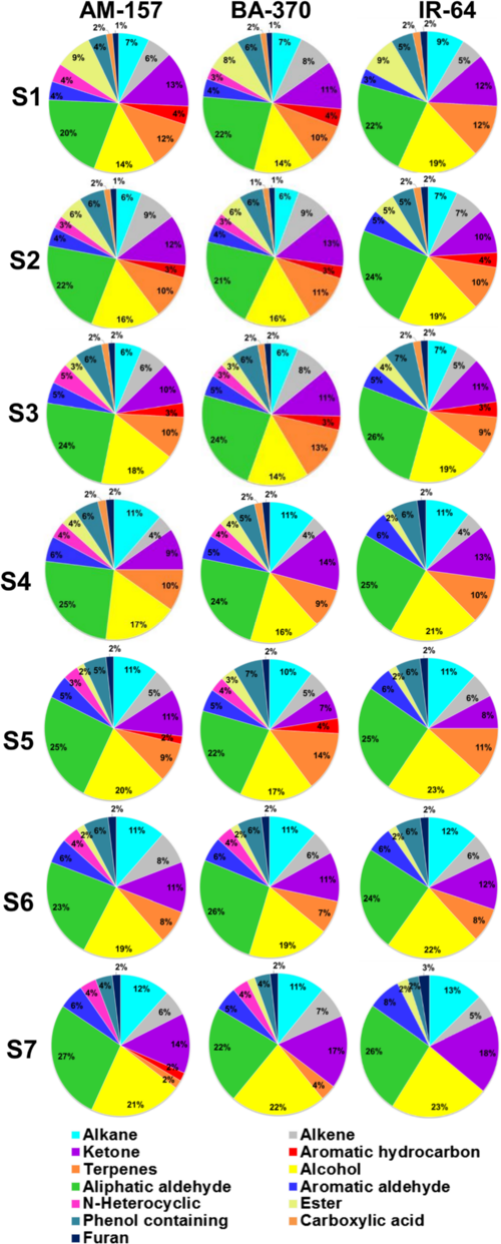
Qualitative analysis of aroma volatiles at 7 developmental stages in 3 rice cultivars (S1- seedling, S2: tillering, S3: booting, S4: flowering, S5: miky grains, S6: dough grains, S7: mature grains)

At seedling stage (S1) in scented category, 72 compounds in BA-370 and 70 in AM-157 belonging to all 13 classes were detected. In IR-64, 58 compounds belonging to 11 classes were detected (Fig. 1, Table 1). VOC’s belonging to the chemical classes aliphatic aldehydes (13–16) and alcohols (10–11) were present in maximum number and contributed 20–22 % and 14–19 % share respectively in total volatile components (Fig. 1). N-heterocyclic (3), aromatic hydrocarbons (3) and other 16 compounds belonging to different classes were present only in scented rice cultivars (Table 1). Among these, 8 compounds (2AP, 2-acetyl-1H-pyrrole, (E)-5-methyl-4-decene, (E)-5-ethyl-6-methyl-3-hepten-2-one, p-xylene, 1-isopropyl-2-methylbenzene, 1-isopropyl-4-methylbenzene and valencen) were found common in both the scented cultivars. Seven compounds (β-ionone, 2-hexyl-1-octanol, 2-hexadecanol, (E,Z)-2,6-nonadienal, 1H-indole, L-limonene and acetic acid, 1,7,7-trimethyl-bicyclo(2,2,1)hept-2-yl, ester) were detected specifically in AM-157 and 7 compounds (allylcyclohexane, (Z)-3-undecene, 6,10-dimethyl-2-undecanone, pinene, (Z)-2-heptenal, methyl 2-aminobenzoate and 3,4-dimethylcyclohexanol) were specific to BA-370.

During S2 stage, 70 compounds were present in BA-370, 68 in AM-157 and 58 in IR-64. In scented rice cultivars 13 chemical classes were detected and 12 classes were found in non-scented type. N-heterocyclic (2AP and 2-acetyl-1H-pyrrole) and other 13 compounds from different classes distinguished scented rice cultivars from non-scented at tillering stage (Table 1, Fig. 1). Among these, 11 compounds were commonly detected in both scented type, camphene and acetic acid, 1,7,7-trimethyl-bicyclo(2,2,1)hept-2-yl, ester were specifically present in BA-370 and 1H-indole and 3,7-dimethyl-1-octanol were in AM-157. Percent share of aldehydes (21–24) and alcohols (16–19) was found maximum at this stage (Fig. 1).

### Reproductive stages (S3: booting and S4: flowering)

In S3 stage number of compounds were decreased (from 72–68 to 63–62) than S1 and S2 in scented rice cultivars. Compounds belonging to alkenes, alkanes, ketones, aromatic hydrocarbons, terpens and ester were decreased at S3 stage (Fig. 1). In S4 stage, number of compounds decreased further i.e., 55 in BA-370, 51 in AM-157 and 48 in IR-64 than earlier stages. Aromatic hydrocarbon class was found totally absent in all three rice cultivars at this stage. The numbers of compounds belonging to classes’ alkenes (6–4 to 2), esters (6–5 to 2) terpenes (8–7 to 5), alcohols (11–10 to 9) aldehydes (16–14 to 11) and phenols (4 to 3) were decreased in this stage than S1 and S2 stages in all three rice cultivars.

### Grain filling stages (S5: milky grain and S6: dough grain)

At S5 stage 58 compounds (BA-370) and 56 compounds (AM-157) belonging to 12 chemical classes and 52 compounds (IR-64) belonging to 10 classes were detected (Fig. 1). N-heterocyclic and aromatic hydrocarbon classes were absent in non-scented rice IR-64. Carboxylic acid class was not detected in any rice cultivar at this stage. Terpenes were present maximum in numbers in BA-370 (8; 14 %) than AM-157 (5; 9 %) and IR-64 (6; 13 %).

At S6 stage, 52 compounds in AM-157, 53 in BA-370 belonging to 11 classes and 50 compounds in IR-64 belonging to 10 classes were detected (Fig. 1). N-heterocyclic class was detected only in scented type and aromatic hydrocarbons and carboxylic acids were absent at S6 stage in all cultivars. The number and percent share of terpenes decreased significantly at S6 stage than S1-S4 stages in all rice cultivars.

### Mature grain stage (S7)

At maturity 51 compounds belonging to 12 chemical classes in AM-157 and 54 compounds belonging to 11 chemical classes in BA-370 were detected. At maturity N-heterocyclic (2), terpenes (3), aromatic hydrocarbons (1), alcohols (4), aliphatic aldehydes (4), ketones (2), alkanes (2), alkenes (3) and phenol (1) were specifically present in scented rice cultivars.

The data analysis revealed that N-heterocyclic compounds (2AP, 2-acetyl-1H-pyrrole and 1H-indole) class was the major distinguishing class between scented from non-scented rice cultivars. The N-heterocyclic compounds are characterized for its role in flavor in many food products (Ho and Carlin 1989; Adams et al. 2001). The odor strength and complexity of these compounds makes them desirable as flavoring ingredients (Vernin and Párkányi 1982; Wang and Kays 2000).

In all identified volatile compounds 40 % compounds were belonging to aldehydes, alcohols and phenol classes across developmental stages in all three rice cultivars. Aldehyde was the second most important class of compound contributing into aroma of rice after N-heterocyclic. Among the detected aldehydes, 10 aliphatic aldehydes [pentanal (strong, acrid odor), heptanal (green, fresh), (Z)-2-heptenal (fatty, green), (E,E)-2,4-decadienal (musty, cooked starch aromas), (E)-2-octenal (nutty, cooked flour), nonanal (grassy, citrus, floral), (E,Z)-2,6-nonadienal (green, metallic), decanal (fatty, fruity), (E,E)-2,4-nonadienal (fatty, metallic) and β-cyclocitral (minty, nutty type odor)] and 3 aromatic aldehydes (benzaldehyde with nutty, sweet tone, phenylacetaldehyde contributing for herbal, floral aroma and vanillin imparting vanilla like flavor) were described earlier as positively associated compounds for rice aroma (Buttery et al. 1986; Buttery et al. 1988; Buttery et al. 1999; Mahattanatawee and Rouseff 2014; Calingacion et al. 2015). However, some compounds like octanal (fatty) and hexanal (green) were found to be imparting off flavor to rice (Bergman et al. 2000; Liyanaarachchi et al. 2014). Alcoholic compounds were not significantly differed throughout the developmental stages (9–12). Percent share of phenol containing compounds reduced gradually from vegetative (6–4 %) and grain filling stages (7–5 %) to maturity (4–2 %) stage (Fig. 1). The aldehydes and alcoholic compounds are synthesized through *lipoxygenase* (*LOX*), or α/β-oxidation from saturated and unsaturated fatty acids (Schreier and Schwab 2002). These are lipid degradation products and contribute for grassy, fatty and soapy flavors to rice.

Percent share of ketone varied from 7 to 18 % through developmental stages and found minimum at milky grain stage (7–11 %) and maximum in mature grains (14–18 %) (Fig. 1). Bryant and Mcclung (2011) reported maximum ketones (10) in freshly harvested aromatic rice cultivars (Aromatic se2, Dellmati, Dellrose, IAC 600, Jasmine 85, JES and Sierra) which were reduced during storage. Terpene compounds were detected more in number during vegetative stage (S1 and S2) and reduced at maturity (Fig. 1). Terpene compounds are most widely occurring naturally in plants and contribute for plant defenses as well as flavor or fragrances in many spices and fruits (Caputi and Aprea 2011). The presence of terpene compounds in rice may contribute for specific aroma during vegetative (S1 and S2) stage but their number decreased greatly at mature stage (S7) indicating lesser contribution towards grain aroma.

Compounds belonging to some classes like alkane, alkene, aromatic hydrocarbons, esters and carboxylic acids have no contribution in rice aroma and they were found common in all cultivars (Wongpornchai et al. 2003; Ajarayasiri and Chaiseri 2008; Bryant and Mcclung 2011; Goufo et al. 2011; Kong and Zhao 2014a).

### Quantitative analysis of aroma volatiles

**Fig. 2:**
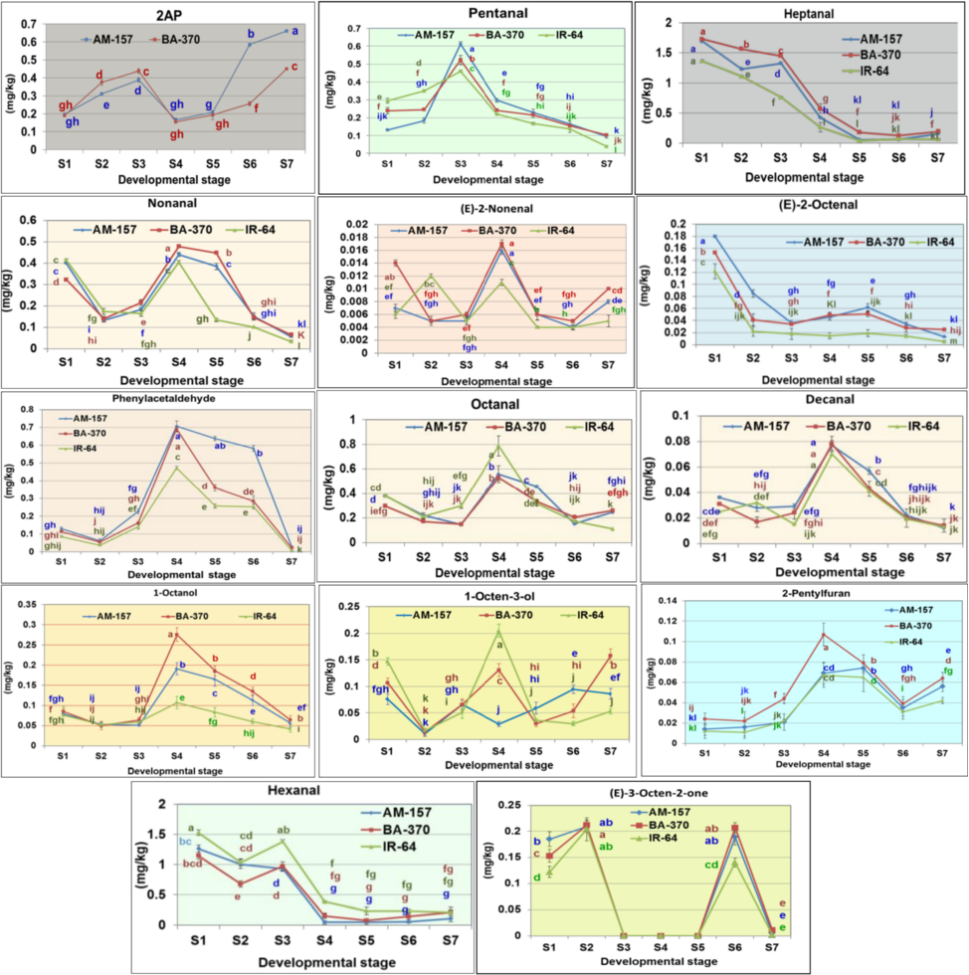
DNMRT analysis of 14 odor active compounds at 7 developmental stages in 3 rice cultivars (S1: seedling, S2: tillering, S3: booting, S4: flowering, S5: miky grains, S6: dough grains, S7: mature grains, difference in lowercase letter indicates significant difference among the mean value presented at *p* = 0.05 level, a-most significant)

Within the developmental stages, nine OACs (2AP, heptanal, (E)-2-octenal, phenylacetaldehyde, 2-pentylfuran, pentanal, nonanal, (E)-2-nonenal and 1-octanol) were significantly elevated in scented rice cultivars over non-scented either in all stages or from S4 to S7 stages indicating their contribution in the aroma of scented rice grains (Fig. 2). Five OAC (2AP, decanal, pentanal, phenylacetaldehyde and hexanal) were accumulated at higher level in AM-157 than BA-370 during S5 to S7 stages and therefore these five compounds might contribute more towards the characteristic flavor of AM-157 rice. (E)-2-Nonenal, nonanal, heptanal, 1-octanol, 1-octen-3-ol and 2-pentylfuran contents were significantly higher in BA-370 during S5 to S7 stages than AM-157 and thus might be contributing in basmati flavor. Accumulation trend of all 14 OACs across seven developmental stages were observed similar in all three rice cultivars.

### Quantification of 2AP

2AP content increased from S1 to S3 stage, decreased during S4 stage and again increased at S6 stage and accumulated to its maximum in S7 stage in both the scented rice cultivars (Fig. 2). In Kyeema rice cultivars, 2AP content was increased three times in mature grains (0.295 mg/kg) than in leaves at tillering stage (0.093 mg/kg) (Wilkie and Wootton 2004). In the present study, similar trend was recorded. 2AP analysis through developmental stages revealed that during booting stage higher level of 2AP was accumulated. Sriseadka et al. (2006) reported that secondary metabolites are mostly produced in rice plant during booting stage. Maximum synthesis of free sugar and starch occurs during booting stage in rice (Togari 1954; Yoshida 1968; Perez et al. 1971). Further, during flowering and grain filling stages the stored starch and free sugar content depleted rapidly where it is utilized for panicle and grain development (Perez et al. 1971). As 2AP is highly volatile and lipophilic in nature, it bounds with lipid molecules and get attached to starch granules (Yoshihashi et al. 2005). The decreased 2AP content from the booting stage until the end of grain development stage is probably due to the translocation of starch bounded 2AP from leaves to grains. 2AP contents reported in mature grains of BA-370 and AM-157 cultivars are in agreement with the previous reports (Mathure et al. 2014). Maximum 2AP accumulation in mature grains can be correlated with higher level of 2AP bound starch content in mature grains (Yoshihashi et al. 2005; Hinge et al. 2016). The present result revealed that enhancement of 2AP content at or after booting stage could increase 2AP accumulation in mature grains. Therefore, agronomical and physiological measures could be taken at or after booting stage to enhance 2AP levels in mature grains.

### Quantification of Aldehydes

Pentanal content initially increased from S1 to S3 stages and further decreased at S4 to S7 stages in both the scented rice cultivars (Fig. 2). Pentanal content was found significantly higher in the scented rice cultivars at S3 to S7 stages (Fig. 2). High level of pentanal in scented rice cultivars at reproductive and grain filling stages contributes to rice aroma. Previously, pentanal has been reported in cooked, brown rice, basmati and other scented rice cultivars and the contents we found in mature grains are in agreement with previous reports (Mathure et al. 2011; Mathure et al. 2014). Pentanal has fruity, green banana-like odor and it is a lipid derivative volatile compound formed from linoleic acid (Suzuki et al. 1999; Yang et al. 2008; Mathure et al. 2014; Hinge et al. 2016). The synthesis of such C5 aldehydes in plants is catalyzed in part by a 13-*lipoxygenase* (*LOX*) (Shen et al. 2014). In the present study, increased pentanal content during early reproductive stages might be due to increased lipid content in rice plant during early reproductive development (Zhang et al. 2010).

Heptanal content was observed significantly more in the scented rice cultivars than non-scented one (Fig. 2). This confirms the contribution of heptanal towards rice aroma. Among the developmental stages, maximum heptanal was recorded at S1 stage and decreased to its minimum at S7 stage in both the scented rice cultivars. In many previous studies heptanal has been identified as a key odorant which gives floral tone to rice scent (Buttery et al. 1988; Yang et al. 2008; Mathure et al. 2014; Nadaf et al. 2016). Heptanal is a fatty acid derived volatile compound associated with green grassy aroma in plants. It is formed by auto-oxidation of palmitoleic acid (Wang et al. 2001). The significantly higher levels of heptanal at S1 stage in both the scented rice cultivars might be due to higher level of fatty acid content during vegetative development. It is also a herbivore induced volatile and more active at early stages of development (Zhou et al. 2003; Aziz et al. 2015; Desurmont et al. 2015). In young leaves of coriander significantly higher level of heptanal was found which decreased further with maturation (Koharai et al. 2006). Moreover, higher level of heptanal was found during vegetative development in tomato (Wang et al. 2001) and early fruit developmental stages of cucumber (Chen et al. 2015).

Nonanal and (E)-2-nonenal contents were significantly increased in scented rice cultivars over non-scented rice at S4 to S7 stages and thus contributed towards the aroma in mature grains (Fig. 2). Higher nonanal level was recorded at S1 and S4 stages and reduced towards S5 to S7 stages in both scented rice cultivars (Fig. 2). Nonanal with fruity, floral aroma is identified as key odorant in various rice cultivars (Yang et al. 2008; Kong and Zhao 2014a; Mahattanatawee and Rouseff 2014; Mathure et al. 2014; Hinge et al. 2016). Nonanal content was elevated in several scented rice cultivars than non-scented one (Yang et al. 2008; Maraval et al. 2008b; Mathure et al. 2011; Mathure et al. 2014). (E)-2-Nonenal content was synthesized to its maximum at S4 stage, reduced at S5 and S6 stages and again increased at S7 stage in both the scented rice cultivars (Fig. 2). (E)-2-Nonenal values in mature grains of scented rice cultivars are in agreement with the previous reports (Widjaja et al. 1996; Mathure et al. 2014). The significantly high levels of (E)-2-nonenal in scented rice cultivars has been reported by Yang et al. (2008); Maraval et al. (2008b). (E)-2-Nonenal having very low odor threshold (0.08 ppb) with fatty, woody, cucumber type aroma was identified as a key odor active compound in AM-157 and BA-370 (Mathure et al. 2014; Hinge et al. 2016). Nonanal, (E)-2-nonenal and other green leaf aldehydes are produced under the *lipoxygenase* (*LOX*) pathway through a complex metabolism in leaves. In the *lipoxygenase* (*LOX*) pathway lipids are first hydrolyzed in free fatty acids by different types of lipases. Then, *LOX* catalyses the stereospecific oxidation of unsaturated free fatty acids. (10E,12E,15Z)-9-hydroperoxy-10,12,15-octadecatrienoic acid (9-HPOT) is produced from the linolenic acid which is transformed to C9-oxo-acids and C9-aldehydes like nonanal and (E)-2-nonenal through the action of *hydroperoxide lyase* (*HPL*) (Taurino et al. 2013; Chen et al. 2015). In plants, nonanal is also synthesized by autoxidation of oleic acid (Tananuwong and Lertsiri 2010). Lipid content including linolenic acid and oleic acid content was found enhanced in flowering panicles and up to 5 days after flowering and reduced further during seed development (Kim et al. 2015). Therefore, there might be higher level of accumulation of these C9 aldehydes during flowering in rice. Nonanal and (E)-2-nonenal were proved as effective antifungal agent and may plays role in protecting valuable reproductive plant organs in rice (Fernando et al. 2005). They were also observed to be induced upon the attack of armyworm and playing important role in volatile-mediated indirect defense mechanism against insects in rice (Yuan et al. 2008). As among the developmental stages, flowering and seedling stages are most sensitive to abiotic and biotic stresses, synthesis of C9 aldehydes (nonanal and (E)-2-nonenal) during this stages might act as an endogenous defense mechanism (Grattan et al. 2002; Desurmont et al. 2015).

The aldehyde (E)-2-octenal (green, fatty, nutty and cooked rice flavor) was accumulated significantly higher in scented rice cultivars than non-scented rice (Fig. 2). It was found maximum at S1 stage and reduced towards reproductive development and maturity (Fig. 2). The values of (E)-2-octenal reported in mature grains of scented rice cultivars are in agreement with the previous report (Mathure et al. 2014). The preeminent content of (E)-2-octenal in scented rice indicated its contributing role in aroma (Maraval et al. 2008b; Mathure et al. 2014). (E)-2-Octenal in rice plant is synthesized from oleic acid and linoleic acid by the action of LOX enzymes (Schwab et al. 2008). (E)-2-Octenal is involved in triggering plant defense mechanism and also shows antimicrobial activity (Noge et al. 2012). The seedling stage is most susceptible to pathogen and insect attack in rice plant and usually affected by infestation of blast, brown spot and stem borers. Higher expression (E)-2-octenal might offer protection to young seedlings of rice against various pathogens and insects (Desurmont et al. 2015).

Phenylacetaldehyde (intense, green, floral aroma) elevated in scented rice cultivars than non-scented rice cultivar (Fig. 2). Stage wise, it increased from S1 to S4 stages and reduced during S5 to S7 stages in both the scented rice cultivars with maximum accumulation at S4 stage (Fig. 2). Phenylacetaldehyde has been reported previously in *O. sativa* subsp. *japonica* cultivar (Lin et al. 2010), Thai fragrant rice (Sunthonvit et al. 2005) and aromatic rice cultivars Yuxiangyouzhan and Nongxiang 18 (Mo et al. 2015). It has been identified as odor active compound in four rice types of *O. sativa* cultivars (Sungeun 2012) and *O. sativa indica* spp (Hinge et al. 2016). Synthesis of phenylacetaldehyde occurs through shikimate/phenylalanine biosynthetic pathway in plants. In rice expression of shikimate kinase gene OsSK1 and OsSK3 is up-regulated specifically during the flowering stage (Kasai et al. 2005). Therefore, there might be maximum synthesis of phenylacetaldehyde at flowering in rice plant. Phenylacetaldehyde is a major volatile compound responsible for aroma of petunia and rose (Kaminaga et al. 2006; Farhi and Dupin 2010). In Arabidopsis maximum amount of phenylacetaldehyde is synthesized during flowering (Gutensohn et al. 2011). Phenylacetaldehyde has been known to possess antimicrobial properties (Berrah and Konetzka 1962) and offer protective role for flowers. In flowering panicles, it is involved in attracting pollinating and predatory insects (Raguso et al. 2003; Jhu et al. 2005).

Octanal (fruity floral) content was elevated in scented rice cultivars during S5 to S7 stages than non-scented rice cultivar (Fig. 2). During development octanal content was increased from S1 to S4 stages and decreased further from S5 to S7 stages. The higher octanal content was previously recorded in basmati than non-aromatic rice cultivars (Yang et al. 2008). Octanal is lipid derivative of oleic acid and imparts off flavor to milled rice (Lam and Proctor 2003; Monsoor and Proctor 2004). The expression of lipoxygenases was found higher during flower and embryo development and moderate in rice leaves (Bañuelos et al. 2008). Thus, there may be higher expression of octanal during flowering in rice. Higher level of octanal was reported as attractants to predatory bugs (Yu et al. 2008). Thus, might be helping rice plant for indirect defense.

Decanal content reduced from S1 to S3 stage and increased significantly at S4 stage. However, in subsequent stages (S5 to S7), it decreased in both scented rice cultivars (Fig. 2). Decanal values reported in mature grains are in agreement with the previous reports (Widjaja et al. 1996; Mathure et al. 2011; Mathure et al. 2014). Though decanal (sweet, citrus, floral type aroma) has been identified as key odorant in many scented rice types like basmati, non-basmati scented, jasmine and tropical aromatic rice cultivars, in present study no significant difference was recorded between scented and non-scented rice cultivars (Yang et al. 2008; Mathure et al. 2014). Similar results were recorded by Maraval et al. (2008a); Maraval et al. (2008b) and Yang et al. (2008). Decanal is a representative of fatty acid-derived volatiles. Similar to other aldehydes, its expression is controlled by *LOX* enzymes. Abundant availability of free fatty acids and lipid molecules and highest expression of *LOX* during flowering may lead to its significantly higher accumulation in this stage. Decanal was identified as one of the important volatiles attracting predatory insects in rice (Fujii et al. 2010).

Hexanal with green grass like aroma showed its maximum content at S1 stage and reduced further at S3 and S4 and at S7 stages in both scented rice cultivars (Fig. 2). Hexanal is reported in many fruits (strawberries, blackberries, mandarin peel, pears and walnut etc.), vegetables (tomatoes, carrot, cucumber etc.) imparting characteristic green/grassy aroma and used as marker for green/grassy aromatics (Abegaz et al. 2004; Chambers and Koppel 2013; Baldwin et al. 2004). It is lipid degradation product and formed by the auto-oxidation of linoleic acid resulting in the development of off and stale flavor (Lam and Proctor 2003; Laohakunjit and Kerdchoechuen 2006). 13-HPOT (9Z,11E,15Z)-13-hydroperoxy-9,11,15-octadecatrienoic acid is produced from linolenic acid and is further metabolized by HPL to form 12-oxo-(Z)-9-dodecenoic acid (a precursor of the traumatin) and (Z)-3-hexenal (Grechkin, 1998). Hexanal has been directly related to oxidative off flavor that increases considerably higher after milling and storage (Buttery et al. 1988; Wongpornchai et al. 2003; Laohakunjit and Kerdchoechuen 2006; Wijerathna et al. 2014). It is also used as indicators of rancidity (Yasumatsu et al. 1966; Champagne et al. 1993). In our studies, hexanal associated with green grassy aroma was significantly higher in leaves at vegetative stage than grain filling and maturity stages. The hexanal accumulation was increased in rice leaves upon pathogen infection and wounding specifically through expression of OsHPL3 gene (Chehab et al. 2006). Highest expression of hexanal during seedling stage might be due to induced defense mechanism which protects young seedlings from pathogen and insect infestation.

### Quantification of alcohols

Significantly higher level of 1-octanol was recorded at S4 to S7 stages in scented rice cultivars than IR-64 (Fig. 2). Highest content of 1-octanol was recorded at S4 stage and reduced further at S5, S6 and S7 stages in both the scented rice cultivars. Earlier, similar patterns of enhanced expression of 1-octanol during flowering stage was reported (Kong and Zhao 2014b). The presence of 1-octanol has been detected in scented rice (Grimm et al. 2011); Yang et al., 2008; Bryant and Mcclung 2011) and identified as odor active compounds (Mahattanatawee and Rouseff, 2014; Kong and Zhao 2014a, b; Hinge et al., 2016). The role of 1-octanol in flowering panicles may be associated with its antifungal and antibacterial activity (Rodov and Nafussi 2011). 1-Octanol has been observed to induce ROS production in elicited and non-elicited rice cells and showed fungistatic activity against the pathogen *Magnaporthe oryzae* (Forlani et al. 2011). Thus maximum accumulation of 1-octanol in flowering panicles might protect the flowers and developing embryos from biotic stress.

Significantly higher quantity of 1-octen-3-ol was recorded at S3 and S5 to S7 stages in scented rice cultivars compared to IR-64 (Fig. 2). Highest level of 1-octen-3-ol was recorded in IR-64 at S4 and S1 stages of development. 1-Octen-3-ol has powerful, sweat, earthy, mushroom type odor with strong herbaceous note. It has been reported as odor contributor in many scented rice cultivars (Mo et al. 2015; Mathure et al. 2014; Yang et al. 2008). The role of 1-octen-3-ol at seedling and flowering stages in rice plant may be associated with plant defense mechanisms. 1-Octen-3-ol was reported as attractant for biting insects, contributes to enhanced plant resistance to the necrotropic fungal pathogen *Botrytis cinerea* by inducing defense signaling cascades and also serves as attractant for fungus-eating beetles (Kanchiswamy et al. 2015).

Ketone (E)-3-octen-2-one was detected only at vegetative and maturity stages and absent at reproductive and milky grain stages in both the scented rice cultivars (Fig. 2). The highest content was recorded at S2 stage followed by S5, S1 and S7 stages. (E)-3-Octen-2-one values recorded in mature grains of scented rice cultivars are in agreement with the previous reports (Maraval et al. 2008b; Luo et al. 2009; Mathure et al. 2014).

### Quantification of furans

2-Pentylfuran was found at significantly elevated level in scented rice cultivars than non-scented rice at all developmental stages (Fig. 2). BA-370 showed elevated level of 2-pentylfuran than AM-157 at all developmental stages. 2-Pentylfuran increased from S1 & S2 stages to S4 stage and gradually decreased at S5 & S6 stages and again increased in S7 stage (Fig. 2). 2-Pentylfuran is a furan compound produced by lipid oxidation and also through maillard reaction. It imparts floral, fruity, nutty and caramel-like aroma to rice. Similar elevated contents of 2-pentylfuran in scented rice cultivars than non-scented rice cultivars were reported by several authors (Sunthonvit et al. 2005; Yang et al. 2008; Maraval et al. 2008b; Luo et al. 2009; Grimm et al. 2011). Its higher concentration in scented rice cultivars and odor active property confirms its role in rice aroma. Its enhanced level at reproductive development could be due to abundant availability of lipid and free fatty acids during flowering stage.

The maximum accumulation of OACs at flowering (nonanal, (E)-2-nonenal, phenylacetaldehyde, octanal, decanal, 1-octanol and 2-pentylfuran) followed by seedling (heptanal, hexanal, (E)-2-octenal and (E)-3-octen-2-one) stages might be due to endogenous developmental regulation of volatile emission. The similar developmental regulation of volatile accumulation in many plant species has been observed. Increased volatile accumulation at vegetative and reproductive development and further remained relatively constant or decreasing trend was reported in many plant species (Vassao et al. 2006; Dudareva et al. 2013). In general, rice seedlings are sensitive to abiotic and biotic stress particularly during seedling, flowering stages (anthesis and fertilization) and to a lesser extent at the preceding stage booting (microsporogenesis) stage than other developmental stages (Grattan et al. 2002). Besides aroma and flavor agents volatile compounds have important contribution in direct and indirect defense mechanism of plant. And thus higher level of expression either at flowering or seedlings stages may be result of ontogeny mechanism of plant defense (Desurmont et al. 2015). Similar results were recorded by Fujii et al. (2010) i.e., relative content of green leaf volatiles (GLVs) was higher in seedlings and flowering panicles than in other plant parts of rice.

### Correlation analysis between OACs

In the correlation analysis of 14 OACs, 35 significant correlations were observed at seven developmental stages in three rice cultivars (Additional file 1: Table S1). Aldehydes showed significant positive correlations with each other and with alcohols and ketones (except hexanal and heptanal). Hexanal, heptanal and pentanal showed significant positive correlation with each other. Octanal, nonanal, decanal, phenylacetaldehyde, 1-octanol and 2-pentlyfuran were positively correlated with each other. 1-octanol levels correlated positively with octanal, nonanal, decanal and phenylacetaldehyde and negatively with hexanal. 2-Pentlyfuran correlated negatively with hexanal, heptanal and (E)-3-octen-2-one. (E)-3-Octen-2-one was positively correlated with heptanal and (E)-2-octenal. 1-Octen-3-ol level was correlated positively with octanal. There was no significant correlation recorded between 2AP and any OAC in both the scented rice cultivars. This indicated that 2AP expression pattern was unique and specific throughout rice plant development. Mathure et al. (2014) observed positive correlation of 2AP with 1-tetradecene and indole and negative correlation with benzyl alcohol in correlation analysis of 23 headspace volatile compounds in mature grains of 91 rice cultivars. But in the present study among developmental stages in three rice cultivars no any positive or negative correlation was observed for 2AP with other OAC. The correlations among the aliphatic aldehydes (heptanal, octanal, nonanal and decanal) recorded in the present study are in agreement with Mathure et al. (2014). These results revealed that 2AP alone has different accumulation behavior and the remaining OACs have similar accumulation trend through out rice plant development.

### PCA analysis between OACs

**Fig. 3:**
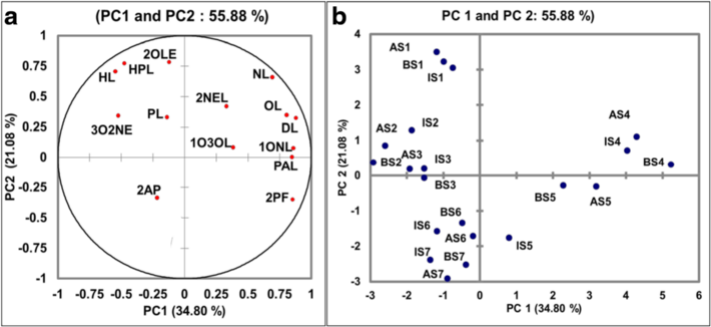
PCA analysis of odor active compounds (**a**) and developmental stages (**b**) of 3 rice cultivars (2AP; 2-acetyl-1-pyrroline, PL; pentanal, HL; hexanal, HPL; Heptanal, OL; Octanal, NL; Nonanal, 2NEL; (E)-2-Nonenal, DL; Decanal, PAL; Phenylacetaldehyde, 1ONL; 1-Octanol, 3O2NE; (E)-3-Octen-2-one, 2PF; 2-Pentylfuran, 2OLE; (E)-2-Octenal, 1O3OL; 1-Octen-3-ol, A in AS1 to AS7; AM-157, B in BS1 to BS7; BA-370, I in IS1 to IS7; IR-64, S1; seedlings, S2; tillering, S3; booting, S4; flowering, S5; milky grains, S6; dough grains, S7; mature grains)

Principle Component Analysis (PCA) between 14 OACs and seven developmental stages in three rice cultivars are summarized in Fig. 3a and b. Fourteen OACs were separated in two principal components at 55.88 % cumulative variance (Fig. 3a). 2AP was separated at negative side of both the PCs and 2-pentylfuran at negative side of PC2 but positive side of PC1. Aldehydes hexanal, heptanal, pentanal, (E)-2-octenal and (E)-3 octen-2-one were separated at negative side of PC1 with 2AP but positive side of PC2. Majority of the aldehydes exhibited significant loadings on positive side of PC2 indicating positive association between them. Among the 14 OACs, 7 compounds viz. 2AP, pentanal, hexanal, heptanal, 1-octen-3-ol, (E)-2-octenal and (E)-3-octen-2-one displayed significant loadings in more than one principle component (Additional file 2: Table S2). This suggests involvement of multiple factors in accumulation of these compounds at the level of synthesis, developmental accumulation and degradation (Yang et al. 2008; Yang et al. 2010; Mathure et al. 2014). Similar results were recorded by Yang et al. (2008); Griglione et al. (2015).

Based on 14 OACs, seven developmental stages could be clearly differentiated (Fig. 3b). Vegetative and booting stages of all three rice cultivars were placed at negative side of PC1 and positive side of PC2 respectively. Flowering stages were isolated from all stages at positive side of both the PC and milky grain stages into positive side of PC1 and negative side of PC2. Dough grain and mature grain stages together placed at negative side of both the PCs. This showed that volatile accumulation pattern through developmental stages was specific for each developmental stage. The variation in OACs was more within the developmental stages than between the cultivars. Hence, developmental stages were separated more clearly than cultivars. Maraval et al. (2008b) reported that OACs could differentiate Thai rice cultivar from Aychade and Fidji cultivars. The aromatic cultivars Hyangmibyeo 1, Hyangmibyeo 2, Royal basmati, Jasmine, Black pigmented and non-aromatic rice were separated based on content of 13 OACs (Yang et al. 2008).

### OACs as biomarker for defining rice cultivars

**Fig. 4:**
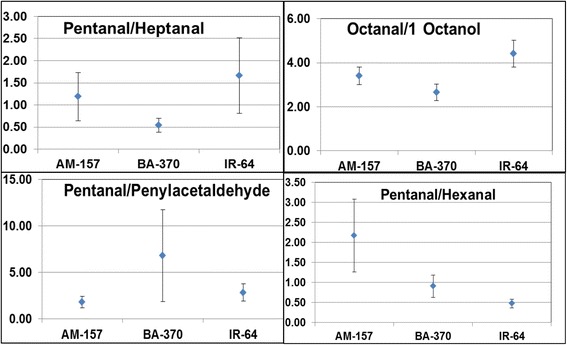
OACs as cultivars specific biomarker identification, average values with standard error for ratio of OACs in respective rice cultivars under study

The volatile compounds expressing in constant ratios across the developmental stages could be considered as biomarkers for defining specific rice cultivar. The octanal/1-octanol content ratio was served as common marker for AM-157 (3.41), BA-370 (2.66) and IR-64(4.41) (Fig. 4). In AM-157 pentanal/phenylacetaldehyde (1.79) ratio was found constant at all developmental stages and could be used to define this cultivar. Pentanal/heptanal ratio remained constant only in BA-370 (0.54) therefore it could be considered as a good indicator for BA-370. Non-scented rice IR-64 represented constant ratio of pentanal/hexanal (0.47) hence could be taken as biomarker specific for IR-64. Such types of biomarkers were previously reported by Griglione et al. (2015) for defining rice cultivars and as indices of aging. They found that heptanal/1-octen-3-ol and heptanal/octanal ratios as aroma quality indices for six Italian rice cultivars. Aroma volatiles were also used as marker to discriminate the rhizobacterial isolates (Deshmukh et al. 2015). Thus, these OACs can be taken as biomarkers for defining the cultivars under study.

### Proline and methylglyoxal content

**Fig. 5:**
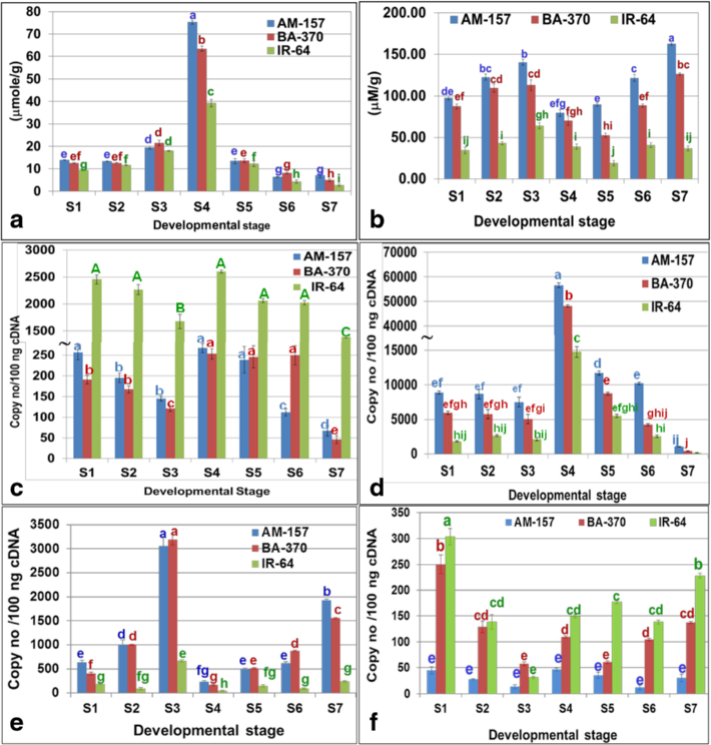
Proline (**a**), methylglyoxal (**b**), *badh*2 (**c**), *P5CS* (**d**), *TPI* (**e**), *GAPDH* (**f**) transcript at 7 developmental stages in 3 rice cultivars. (S1; seedlings, S2; tillering, S3; booting, S4; flowering, S5; milky grains, S6; dough grains, S7; mature grains. *badh*2; betaine aldehyde dehydrogenase 2, *TPI;* triose phosphate isomerase*, GAPDH;* glyceraldehyde-3-phosphate dehydrogenase, *P5CS;* Δ*1-Pyrolline-5-carboxylic acid synthetase,* Difference in lowercase letter indicates significant difference among the mean value, A or a-most significant)

Proline content was recorded higher in scented rice cultivars than non-scented rice cultivar IR-64 at all developmental stages (Fig. 5a). Highest proline content was recorded at S4 stage in both the scented rice cultivars (Fig. 5a). Proline content remained constant during vegetative development and increased significantly at flowering and reduced at grain filling and maturity stages in both scented rice cultivars. Proline levels were in agreement with those reported by Kaikavoosi et al. (2015). Proline was identified as a precursor of 2AP (Suprasanna et al. 1998; Suprasanna et al. 2002; Yoshihashi et al. 2002a) and increase in free proline content leads in increased 2AP content in several scented rice cultivars (Poonlaphdecha et al. 2012; Mo et al. 2015). Proline accumulation in rice plant was determined by developmental stage and type of plant organs (Nanjo et al. 1999; Maggio et al. 2002). In flowering stage, the panicles showed highest proline accumulation, which contributes in flower and embryo development. Several studies indicated that proline plays an important role in reproductive development especially at flower development and serving as a readily accessible source of energy in pollen (Lehmann et al. 2010). Analysis of the free amino acids in different tissues revealed that proline content was 60 times higher in pollen than in any other organ (Szabados and Savouré 2010). Thus in reproductive developmental stages proline plays major role. The left over proline through primary metabolism might be utilized for 2AP synthesis; hence, in spite of level of proline, 2AP contents might be low in flowering panicles.

Methylglyoxal was accumulated at significantly higher level in scented rice cultivars than IR-64 (Fig. 5b). At the S7 stage, highest MG content was recorded followed by S3 stage in AM-157. MG contents were positively correlated with 2AP accumulation in both the scented rice cultivars. Increase in MG content leads in increased 2AP content in aromatic rice cultivars Tainung 71 and 72 and in aromatic soybean (Huang et al. 2008; Wu et al. 2009; Szabados and Savouré 2010). The methylglyoxal contents recorded in the present study are comparable with earlier reports (Yadav et al. 2005; Huang et al. 2008; Hossain et al. 2009; Kumar and Yadav 2009). This confirmed the role of methylglyoxal as a precursor in 2AP accumulation.

### Gene expression analysis

Expression of *badh*2 was found to be 9 to 30 fold reduced in both the scented rice cultivars than non-scented IR-64 at all developmental stages (Fig. 5c). At S7 and S3 stages, 1.7 to 5.56 fold lower transcript was recorded than S1, S2, S4 to S6 stage in both the scented rice cultivars. Significantly, higher level of 2AP was accumulated in the S7 and S3 stages of both scented. This indicated that *badh*2 expression is negatively associated with 2AP accumulation in scented rice. 2AP accumulation is regulated by *badh*2 transcript (Vanavichit et al. 2008). The down regulation of *badh*2 gene was also previously reported in aromatic rice cultivars (Chen et al. 2008; Niu et al. 2008; Vanavichit et al. 2008).

*P5CS* gene expression was 3 to 5 fold higher in both the scented rice cultivars compared to IR-64 at all developmental stages (Fig. 5d). Highest transcript was recorded at S4 stage in both the scented rice followed by S5 stage in AM-157. *P5CS* expression was low at S7 stage than other stages. *P5CS* expression was not significantly different among S1, S2 and S3 stages in scented rice cultivars. Positive association of 2AP accumulation with *P5CS* expression has been reported earlier (Wu et al. 2009; Huang et al. 2008). Up regulation of expression of Δ*1-pyrroline-5-carboxylate synthetase* (*P5CS*) in aromatic rice might increase the level of Δ1-pyrroline-5-carboxylic acid (P5C) and thus more 2AP accumulation (Huang et al. 2008). Proline has been also identified as important precursor of 2AP and proline synthesis in plant is mediated largely by *P5CS* enzyme (Schieberle 1990; Romanczyk et al. 1995; Suprasanna et al. 2002; Yoshihashi et al. 2002b; Thimmaraju et al. 2005). Proline is further metabolized to Δ1-pyrroline that reacts with methylglyoxal to form 2AP (Huang et al. 2008). Kaikavoosi et al. (2015) reported more than 2 fold enhancements in 2AP content after overexpression of *P5CS* in transgenic calli, vegetative plant parts and seeds of AM-157 and Indrayani rice cultivars over control. Significantly, higher level of *P5CS* in scented rice cultivars over non-scented in present study confirms the role of *P5CS* in 2AP accumulation. However across the developmental stages *P5CS* expression and 2AP accumulation were not correlated positively that might be due to involvement of *P5CS* and proline in plant development as well as 2AP accumulation. The *P5CS* dependent proline synthesis from glutamate was associated with flower development (Lehmann et al. 2010). In *Arabidopsis P5CS*1 was present exclusively in anthers, whereas *P5CS*2 was found in inflorescence meristems, flower primordia and flower buds (Székely et al. 2008). Both the *P5CS* genes in *Arabidopsis* were involved in controlling flowering (Mattioli et al. 2008; Mattioli et al. 2009; Mattioli et al. 2012). Overexpression of *P5CS* leads to enhance proline accumulation as well as flower development in transgenic plants (Kishor et al. 1995).

Triose phosphate isomerase (TPI) gene expression was found significantly higher (2 to 10 fold) in both the scented rice cultivars than IR-64 at all developmental stages (Fig. 5e). Significantly higher TPI transcript was recorded in S3 stage followed by S7, S2, and S6 stages of scented rice cultivars. 2AP accumulation was higher in S7 and S3 stages of scented rice cultivars. TPI activity controls MG level in plants. MG is produced from non-enzymatic elimination of phosphate from glyceraldehyde-3-phosphate (GAP) and dihydroxyacetone phosphate (DHAP). The inter conversion of DHAP and GAP is catalyzed by a crucial glycolytic enzyme, triose phosphate isomerase (TPI) (Sharma et al. 2012). The TPI expression has been well studied in rice (Sharma et al. 2012) and it was observed that its expression increased during reproductive developmental stages and at grain filling to maturity stages in rice cultivar IR-64. Similar results were observed in the present study. Increased TPI expression led to increase MG concentration; similar results were recorded earlier where increase in MG concentration correlated positively with TPI activity in rice (Sharma et al. 2012).

The *glyceraldehyde-3-phosphate dehydrogenase* (*GAPDH*) expression was found lower in scented rice cultivars than non-scented rice IR-64 at all developmental stages (Fig. 5f). The expression of *GAPDH* was lowest in S3 stage than other developmental stages in both the scented rice cultivars. The 2AP accumulation and *GAPDH* expression were negatively associated and reduced expression of *GAPDH* may enhance 2AP accumulation in scented rice cultivars. In the present study *GAPDH* expression varied up to five fold between cultivars and up to 2 fold within cultivars at different developmental stages (Fig. 5f). The range of transcript values of all genes are comparable with previous reports (Whelan et al. 2003; Ohdan et al. 2005; Dhanasekaran et al. 2010). Though *glyceraldehyde-3-phosphate dehydrogenase* (*GAPDH*) has been used as housekeeping gene, some recent studies showed that *GAPDH* expression was not stable across different tissue and experimental treatments (Jain et al. 2006; Tong et al. 2009; Li et al. 2010; Ling et al. 2014; Su et al. 2014) in rice. Up to two-fold variation in *GAPDH* expression were observed between samples in six cultivars of rice (Kim et al. 2003), while among two cultivars of petunia (*Petunia hybrida* L.), the difference in stability between them was four-fold (Mallona et al. 2010). Thus the present results are in agreement with these reports.

### PCA and correlation analysis between 2AP, gene expression and metabolites

**Fig. 6:**
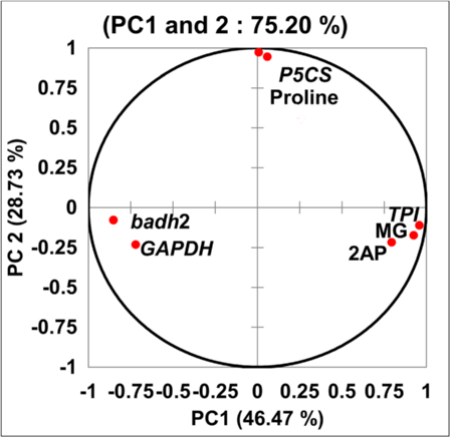
PCA analysis of 2AP, proline, MG, *badh*2, *TPI, GAPDH* and *P5CS* at 7 developmental stages in 3 rice cultivars (*badh*2: *betaine aldehyde dehydrogenase* 2; *TPI : triose phosphate isomerase;, GAPDH : glyceraldehyde-3-phosphate dehydrogenase* and *P5CS:* Δ*1-Pyrolline-5-carboxylic acid synthetase*)

The PCA analysis clearly distinguished the parameters under study into 2 PCs with 75.20 % cumulative variance (Fig. 6). 2AP, MG and TPI were placed in positive side of PC1 confirming their close association. Proline and *P5CS* were placed at positive side of PC1 with 2AP indicating there positive contribution in 2AP accumulation. *GAPDH* and *badh*2 were separated opposite to 2AP at negative side of PC1 and PC2. This confirmed there negative correlation in 2AP accumulation. Similar separation of 2AP and proline was reported by Poonlaphdecha et al. (2012) at different developmental stages in Aychade rice cultivar against salt stress.

The results of correlation analysis between 2AP, gene expression and metabolites are depicted in Table 2. Significant negative correlation between *badh*2 and 2AP accumulation confirmed its recessive nature in scented rice cultivars. The negative correlation of 2AP with *badh*2 expression also reported previously in many scented rice cultivars (Chen et al. 2008; Niu et al. 2008; Vanavichit et al. 2008; Chen et al. 2012). There is no significant positive correlation recorded for 2AP with proline content and *P5CS* expression across developmental stages. It might be due to developmental regulation of proline synthesis in plants (Lehmann et al. 2010). Significant positive correlation of 2AP with MG confirms that MG is one of the precursors in 2AP biosynthesis. This is in agreement with reports of Huang et al. (2008) and Wu et al. (2009). The positive correlation of *TPI* with MG and its negative correlation with *GAPDH* showed *TPI* and *GAPDH* as a major gene controlling methylglyoxal level in rice. Sharma et al. (2012) observed increased *TPI* activity with increase in MG concentration in rice. The role of *TPI* and *GAPDH* in biosynthesis of MG and 2AP has been reported earlier in aromatic rice (Huang et al. 2008) and scented soybean (Wu et al. 2009).

**Table 2:**
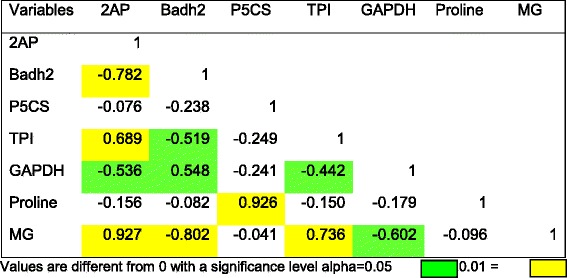
Correlation analysis of 2AP, Proline, MG, content with Badh2, P5CS, TPI and GAPDH expression at various developmental stages in three rice cultivars

### Mechanism of 2AP accumulation in rice grains

**Fig. 7:**
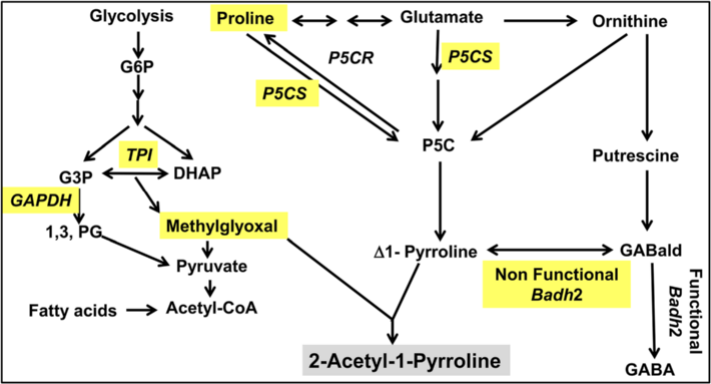
Biosynthesis pathway of 2AP in scented rice cultivars (G6P: Glucose 6 Phosphate; P5CS: Δ*1Pyrroline-5 carboxylate synthetase*; *P5CR: Pyrroline-5 carboxylate reductase*; GABald: γ- Amino Butyraldehyde; GABA: γ- Amino Butyric acid. *TPI* : *triose phosphate isomerase*; *badh*2: *betaine aldehyde dehydrogenase*; *GAPDH* : *glyceraldehyde-3-phosphate dehydrogenase*; 1,3, PG : 1,3-bisphosphoglyceric acid; DHAP: Dihydroxyacetone phosphate; Acetyl CoA : Acetyl coenzyme A)

There are two mechanisms proposed for accumulation of 2AP in mature grains. In one mechanism, 2AP is synthesized in leaves and stem sheaths and transported to mature grains. As per the another one, proline translocates from leaves into grains and 2AP synthesis occurs in grains (Poonlaphdecha et al. 2012; Mo et al. 2016; Poonlaphdecha et al. 2016). In the present study, maximum 2AP was recorded in mature grains, with less proline and low *P5CS* expression as compared to other developmental stages. This suggests the existence of former mechanism in the cultivars under study. This mechanism of 2AP accumulation in mature grains is supported by following reports. Proline level was not significantly affected in grains by salinity or drought stress although these grains contained a significantly higher amount of 2AP (Yoshihashi et al. 2002a; Yoshihashi et al. 2002b; Gay et al. 2010; Summart and Thanonkeo 2010; Poonlaphdecha et al. 2012). Chen et al. (2012) demonstrated that down regulation of BADH2 using amiRNA driven by the maize ubiquitin promoter increased proline and 2AP content in leaf and grains of the transgenic rice. But when the same amiRNA was driven by the endosperm-specific promoter GluC, no effect on BADH2 expression was observed and the 2AP content in grains did not differ from wild-type grains. Among the studied metabolites; enzymes and genes contributing in 2AP biosynthesis are highlighted in Fig. 7.

## Conclusions

The study indicated that volatilome of scented rice cultivars was more complex than non-scented rice cultivar. N-heterocyclic (2AP, 2-acetyl-1-pyrrole and indole) was the major distinguishing class between scented from non-scented rice cultivars. 2AP and other 13 compounds were detected specifically in scented rice cultivars. Maximum number of compounds were synthesized at seedling stage and decreased gradually at reproductive and maturity. The seedling stage is an active phase of development where maximum number green leaf volatiles were synthesized which could be acting as defense molecules for protection of young plant parts. Among the 14 OACs, 10 OACs were accumulated at higher concentrations significantly in scented rice cultivars and contribute in the aroma. Among the scented rice cultivars, specific set of OACs could distinguish BA-370 and AM-157 and can be taken as Basmati and Ambemohar flavor types. 2AP content was highest in mature grains followed by the booting stage. The maximum accumulation of OACs at flowering stages might be due to endogenous developmental regulation of volatile emission. Gene expression analysis revealed that reduced expression *badh*2 and *GAPDH* and elevated level of *TPI* and *P5CS* transcript enhances 2AP accumulation.

Most diverse compounds were synthesized at seedling stage and OACs were accumulated more at flowering followed by seedling stage. Distinct accumulation pattern exists for 2AP and other aroma volatiles at various developmental stages. OACs were identified as biomarkers for defining rice cultivars and selective OACs could be enhanced for inducing basmati or ambemohar type flavor. By designing proper management practices, 2AP contents at booting stage could be maintained to further enhance aroma in mature grains.

## Methods

### Plant material

The seeds of scented rice cultivars Ambemohar-157 (AM-157) and Basmati-370 (BA-370) were procured from Rice Research Station, Vadgaon Maval, Maharashtra, India. The seeds of non-scented rice cultivar IR-64 were procured from Balashaeb Sawant Kokan Krishi Vidyapeeth (BSKKV), Dapoli, Maharashtra, India. The seeds of three rice cultivars were sown in pots under greenhouse following completely randomized block design in three replications at Vilasrao Deshmukh College of Agricultural Biotechnology (VDCOAB), Latur, Maharashtra, India. The four seedlings were planted into each pot and kept 30 pots per cultivar. Routine cultivation practices were adapted thought growing season of rice (Hukkeri 1981; Kediyal and Dimri 2009). Seven developmental stages selected for study are seedling (S1), tillering (S2), booting (S3), flowering (S4), milky grains (S5), dough grains (S6) and mature grains (S7). Leaf samples were collected during S1 to S3 stages, panicles during S4 to S6 and mature grains at S7 stages. Every sampling was done at morning 10.00 am and samples of nine randomly selected plants (3 from each replication) of each cultivars were pulled together. Further samples were powdered using liquid nitrogen and distributed into 3 aliquots and stored at −80 °C, one used for volatile analysis, 2^nd^ for RNA extraction and 3^rd^ for other metabolite analysis. Three biological and two technical replicates were used for biochemical and molecular analysis of each sample.

### Extraction, identification and quantitation of volatiles

Volatile compounds were extracted using head-space solid phase micro extraction (HS-SPME) method and 1 cm long fibers coated with Carboxen/Divinyl-benzene/Poly-dimethyl-siloxane (CAR/DVB/PDMS) with a manual holder (Wongpornchai et al. 2003; Laguerre et al. 2007; Grimm et al. 2011). 4 ml screw top vials (15 × 45 mm) with PTFE (Polytetrafluoroethylene) silicon septa (Chromatography research supplies, Louiseville, KY, USA) were used for analysis. The vials were heated in oven at 150 °C for 1 h prior to use for elimination of unintended volatile compounds. The optimized HS-SPME conditions were used for volatile analysis of leaf (Hinge et al. 2016) and mature grain (Mathure et al. 2011) samples.

Separation and analysis of volatile compounds was done using HS-SPME coupled with GC-MS (Varian 430-GC and 210-MS, Japan) with a factor four capillary column VF5-MS (30 m × 0.25 mm × 0.25 μm) (Varian, Inc., Palo Alto, CA) of 5 % diphenyl, 95 % dimethyl polysilosane. The research grade helium (99.999 %) was used as the carrier gas under a constant flow of 28.6 cm/s (1 ml/min). Volatiles were extracted and concentrated by using preconditioned SPME fiber at 250 °C for 30 min attached to the SPME manual holder (57330-U) (Supelco, Bellefonte, PA, USA). The SPME fiber was desorbed for 5 min in GC injector having temperature at 260 °C. Optimum performance of SPME fiber was monitored after every ten extractions with the standard fiber maintained separately. The fiber showing comparable performance was continued in further analysis. Blanks were run following every fourth sample as a control. The GC oven program was optimized for the separation of 2AP from seed and leaf samples. Initially, oven temperature was kept at 45 °C for 1 min and ramped to 55 °C at the rate 5 °C/min, then at the rate of 9 °C/min up to 120 °C, further ramped to 240 °C at the rate 15 °C/min with final hold of 1 min. The total GC cycles consisted of 19.22 min runs and 1 min re-stabilization time. Blank run of GC-MS was also performed after every sixth samples to remove traces (if any) from earlier runsin GC column. The injector temperature was 260 °C and the transfer line was held at 230 °C. The detection was performed by a Saturn III mass spectrometer in the EI mode (ionization energy, 70 eV; source temperature, 180 °C). The MS was operated in the scan mode from m/z 35 to 275. Identification and quantification of 2AP and other volatiles were done at selected seven developmental stages as described previously (Hinge et al. 2016). The comparative GC-chromatograms for three rice cultivars at seven developmental stages are shown in Additional file 3: Figure S1. During the analysis, 14 volatile compounds were identified as odor active compounds (OACs) that were analyzed quantitatively in the seven developmental stages in all three rice cultivars. This includes 2AP, 8 aliphatic aldehydes (pentanal, hexanal, heptanal, octanal, (2E)-2-nonenal, nonanal, decanal and (E)-2-octenal), 1 aromatic aldehyde (phenylacetaldehyde), 2 alcohols (1-octanol and 1-octen-3-ol), 1 furan (2-pentylfuran), and 1 ketone (E)-3-octen-2-one).

### RNA extraction and cDNA synthesis

RNA was extracted from the leaves, panicles of rice cultivars under study using TRIzol reagent following the manufacturer’s protocol (Invitrogen, Carlsbad, CA) and treated with DNase (Fermentas, Germany). RNA was extracted from ~100 mg mature grains using extraction buffer (100 mMTris-HCl, 150 mM LiCl2, 50 mM EDTA, 1.5 % SDS, 1.5 % β-mercaptoethanol), and phenol/chloroform extraction followed by TRIzol reagent. RNA concentration in samples was determined using a Nanodrop ND-1000 (Nanodrop Technologies, Wilmington, DE, USA). First-strand cDNA synthesis was done using ~1 μg total RNA from each sample and RevertAid first strand cDNA synthesis kit (Thermo Scientific). cDNA was then aliquoted and stored at −80 °C.

### Quantitative Real-Time PCR (qRT-PCR)

For performing qRT-PCR selected genes (*badh*2, *P5CS*, *TPI*, *GAPDH* and EF1α as housekeeping gene) were amplified from cDNA of leaves of BA-370 using gene specific primers and cloned into pJET cloning vector (Life Technologies, Carlsbad, CA, USA). For *badh*2 gene, primers (forward: TGTGCTAAACATAGTGACTGGA, reverse: CTTAACCATAGGAGCAGCT) were designed to target exon 6 and 7 region of *badh*2 gene [Genebank: FJ70385]. For GAPDH primers (forward: ATGGCGAAGATTAAGATCGGGAT, reverse: CACAGTGTCATACTTGAACA) were designed based reference gene sequence [Genebank:GQ848049]. Primers for *P5CS*, *TPI* and EF1α were used as reported earlier by Yooyongwech et al. (2012), Sharma et al. (2012) and Jain et al. (2006). The purified linear plasmid containing gene of interest was used as standard DNA for developing standard curves (Taverniers et al. 2004; Ohdan et al. 2005) for each gene under study (Additional file 4: Figure S2). The real-time quantitative PCR was carried out in a total volume of 25 μl containing12.5 μl VeriQuest™ Fast SYBR® Green qPCR Master Mix with ROX (2X), 1 μl of each primer (10 pmol/μl), 1.0 μl of cDNA, and 9.5 μl DDH2O. Thermal cycling consisted of a hold at 94 °C for 2 min, followed by 40 cycles of 94 °C for 30 s, 55 °C for 30 s, and 72 °C for 30s. The PCR reactions were performed on the Mastercycler® eprealplex PCR system (Eppendorf, Hamburg, Germany) in triplicate. Primer specificity and gene specific amplification were confirmed with melt curve analysis. Each primer pair has specific TM and only single peak detected in melt curve analysis (Additional file 5: Figure S3). Three biological replicates were used for each sample and reaction for each test sample was repeated three times. Mean ct values were used quantification. Expression analysis in terms of copy number for *badh*2 (*R*^*2*^ = 0.996, e = 1.16); *P5CS* (*R*^*2*^ = 0.983, e = 1.14); *TPI* (*R*^*2*^ = 0.992, e = 1.12); *GAPDH* (*R*^*2*^ = 0.990, e = 1.09) and EF1aplha (*R2* = 0.995, e = 1.11) as a control gene were calculated at selected developmental stages from three rice cultivars. The transcript abundance was expressed as copy number/100 ng of cDNA.

### Proline and methylglyoxal estimation

Free proline and methylglyoxal content were estimated following the method of (Bates et al. 1973) and (Yadav et al. 2005), respectively. Standard curve for proline (Y = 0.011X + 0.0064; R^2^ = 0.988) and methylglyoxal (Y = 0.011X + 0.0064; *R*^*2*^ = 0.988) were developed using standard proline and methylglyoxal (Himedia, India). The concentration of extracted proline and methylglyoxal were extrapolated using these standard curves. The estimation of both was performed in triplicates using three biological replicates.

### Statistical analysis

Descriptive analysis of volatiles was performed to determine the mean, standard deviation, standard error and % coefficient of variation (CV %). Duncan’s multiple range tests were performed on the mean values of each OAC, gene transcripts and other metabolites at various developmental stages in three rice cultivars to identify significant variation between means. Principle component analysis (PCA) was performed to study variations in the composition of 14 OACs, gene transcripts and other metabolites among seven developmental stages in three rice cultivars using XLSTAT software (Version 2014, Addinsoft™).

## Abbreviations

2AP, 2-acetyl-1-pyrroline; AM-157, Ambemohar-157; amiRNA, artificial micro RNA; BA-370, Basmati-370; *badh*2, *betaine aldehyde dehydrogenase* 2; DHAP, dihydroxyacetone phosphate; GAP, glyceraldehyde-3-phosphate; *GAPDH*, *glyceraldehyde-3-phosphate dehydrogenase*; HPL, hydroperoxide lyase; LOX, lipoxygenase; MG, methylglyoxal; OACs, odor active compounds; *P5CS*, Δ1-*Pyrolline-5-carboxylic acid synthetase*; PCA, principle component analysis; qRT-PCR, Quantitative Real-Time PCR; *TPI*, *triose phosphate isomerase*; VOC’s, volatile organic compounds

## Additional files

Additional file 1: Table S1. Correlation analysis of 14 odor active compounds at various developmental stages in three rice cultivars (2AP; 2-acetyl-1-pyrroline, PL;pentanal, HL-hexanal, HPL; Heptanal, OL; Octanal, NL; Nonanal, 2NEL; (E)-2-Nonenal, DL; Decanal, PAL; Phenylacetaldehyde, 1ONL; 1-Octanol, 3O2NE; (E)-3-Octen-2-one, 2PF; 2-Pentylfuran, 2OLE; (E)-2-Octenal, 1O3OL; 1-Octen-3-ol). (DOCX 25 kb) Additional file 2: Table S2. PCA analysis of 14 odor active compounds at various developmental stages in three rice cultivars (2AP; 2-acetyl-1-pyrroline, PL;pentanal, HL-hexanal, HPL;Heptanal, OL;Octanal, NL;Nonanal, 2NEL; (E)-2-Nonenal, DL; Decanal, PAL; Phenylacetaldehyde, 1ONL; 1-Octanol, 3O2NE; (E)-3-Octen-2-one, 2PF; 2-Pentylfuran, 2OLE; (E)-2-Octenal, 1O3OL; 1-Octen-3-ol). (DOCX 15 kb) Additional file 3: Figure S1. Comparative GC chromatograms at 7 developmental stages in 3 rice cultivars, S1; seedlings, S2; tillering, S3; booting, S4; flowering, S5; milky grains, S6; dough grains, S7; mature grains. (TIFF 9631 kb) Additional file 4: Figure S2. Standard curves developed for *badh*2, *P5CS*, *TPI*, *GAPDH* and EF1α genes. (TIFF 1042 kb) Additional file 5: Figure S3. Melt curves of *badh*2, *P5CS*, *TPI*, *GAPDH* and *EF* 1α genes. (TIFF 2645 kb)
