# Supplementary material for: Aroma volatile analyses and 2AP characterization at various developmental stages in Basmati and Non-Basmati scented rice (Oryza sativa L.) cultivars
Source: Rice (N Y). 2016 Aug 5;9:38. doi: 10.1186/s12284-016-0113-6 (PMC4975739; doi:10.1186/s12284-016-0113-6)
Supplement: Additional file 1: Table S1. — Correlation analysis of 14 odor active compounds at various developmental stages in three rice cultivars (2AP; 2-acetyl-1-pyrroline, PL;pentanal, HL-hexanal, HPL; Heptanal, OL; Octanal, NL; Nonanal, 2NEL; (E)-2-Nonenal, DL; Decanal, PAL; Phenylacetaldehyde, 1ONL; 1-Octanol, 3O2NE; (E)-3-Octen-2-one, 2PF; 2-Pentylfuran, 2OLE; (E)-2-Octenal, 1O3OL; 1-Octen-3-ol). (DOCX 25 kb) [file 12284_2016_113_MOESM1_ESM.docx]

**Table S1 Correlation analysis between 14 OACs at 7 developmental stages in 3 rice cultivars.**

| Variables | 2AP | PL | HL | HPL | OL | NL | 2NEL | DL | PAL | 1ONL | 3O2NE | 2PF | 2OLE | 1O3OL |
| --- | --- | --- | --- | --- | --- | --- | --- | --- | --- | --- | --- | --- | --- | --- |
| 2AP | **1** |  |  |  |  |  |  |  |  |  |  |  |  |  |
| PL | -0.093 | **1** |  |  |  |  |  |  |  |  |  |  |  |  |
| HL | -0.213 | **0.525** | **1** |  |  |  |  |  |  |  |  |  |  |  |
| HPL | 0.037 | **0.468** | **0.823** | **1** |  |  |  |  |  |  |  |  |  |  |
| OL | -0.355 | -0.038 | -0.130 | -0.142 | **1** |  |  |  |  |  |  |  |  |  |
| NL | -0.292 | 0.130 | 0.085 | 0.195 | **0.736** | **1** |  |  |  |  |  |  |  |  |
| 2NEL | -0.055 | 0.031 | 0.051 | 0.207 | **0.445** | **0.406** | **1** |  |  |  |  |  |  |  |
| DL | -0.302 | 0.059 | -0.278 | -0.131 | **0.808** | **0.778** | **0.439** | **1** |  |  |  |  |  |  |
| PAL | -0.090 | 0.019 | **-0.494** | -**0.437** | **0.585** | **0.595** | 0.149 | **0.776** | **1** |  |  |  |  |  |
| 1ONL | -0.078 | -0.112 | **-0.474** | -0.312 | **0.556** | **0.702** | 0.214 | **0.749** | **0.813** | **1** |  |  |  |  |
| 3O2NE | 0.066 | -0.208 | 0.321 | **0.395** | -0.342 | -0.172 | -0.058 | -0.327 | -0.295 | -0.274 | **1** |  |  |  |
| 2PF | -0.064 | -0.216 | **-0.652** | **-0.597** | **0.541** | **0.372** | 0.110 | **0.622** | **0.606** | **0.719** | **-0.665** | **1** |  |  |
| 2OLE | -0.028 | -0.060 | **0.532** | **0.648** | 0.105 | **0.482** | 0.208 | 0.075 | -0.117 | 0.043 | **0.399** | -0.328 | **1** |  |
| 1O3OL | 0.038 | -0.120 | -0.003 | -0.074 | **0.518** | 0.303 | 0.216 | 0.222 | 0.157 | 0.163 | -0.289 | 0.350 | 0.097 | **1** |

**Values are different from 0 with a significance level alpha=0.05 and 0.01 =**

(2AP; 2-acetyl-1-pyrroline, PL;pentanal, HL-hexanal, HPL;Heptanal, OL;Octanal, NL;Nonanal, 2NEL; (E)-2-Nonenal, DL;Decanal, PAL; Phenylacetaldehyde, 1ONL; 1-Octanol, 3O2NE; (E)-3-Octen-2-one, 2PF; 2-Pentylfuran, 2OLE; (E)-2-Octenal, 1O3OL; 1-Octen-3-ol)
