# Supplementary material for: Aroma volatile analyses and 2AP characterization at various developmental stages in Basmati and Non-Basmati scented rice (Oryza sativa L.) cultivars
Source: Rice (N Y). 2016 Aug 5;9:38. doi: 10.1186/s12284-016-0113-6 (PMC4975739; doi:10.1186/s12284-016-0113-6)
Supplement: Additional file 2: Table S2. — PCA analysis of 14 odor active compounds at various developmental stages in three rice cultivars (2AP; 2-acetyl-1-pyrroline, PL;pentanal, HL-hexanal, HPL;Heptanal, OL;Octanal, NL;Nonanal, 2NEL; (E)-2-Nonenal, DL; Decanal, PAL; Phenylacetaldehyde, 1ONL; 1-Octanol, 3O2NE; (E)-3-Octen-2-one, 2PF; 2-Pentylfuran, 2OLE; (E)-2-Octenal, 1O3OL; 1-Octen-3-ol). (DOCX 15 kb) [file 12284_2016_113_MOESM2_ESM.docx]

**Table S2 : PCA analysis of 14 odor active compounds at various developmental stages in three rice cultivars.**

| Odor active compounds | F1 | F2 | F3 | F4 | F5 |
| --- | --- | --- | --- | --- | --- |
| 2AP | 0.047 | 0.113 | 0.053 | 0.303 | **0.407** |
| PL | 0.019 | 0.110 | 0.301 | **0.418** | 0.095 |
| HL | 0.299 | **0.501** | 0.087 | 0.025 | 0.016 |
| HPL | 0.226 | **0.600** | 0.057 | 0.001 | 0.037 |
| OL | **0.648** | 0.122 | 0.014 | 0.003 | 0.079 |
| NL | **0.479** | 0.435 | 0.000 | 0.000 | 0.009 |
| 2NEL | 0.108 | 0.177 | 0.058 | 0.078 | 0.000 |
| DL | **0.771** | 0.105 | 0.001 | 0.014 | 0.008 |
| PAL | **0.716** | 0.000 | 0.026 | 0.018 | 0.092 |
| 1ONL | **0.735** | 0.006 | 0.023 | 0.001 | 0.107 |
| 3O2NE | 0.274 | 0.117 | **0.321** | 0.072 | 0.011 |
| 2PF | **0.724** | 0.122 | 0.021 | 0.001 | 0.001 |
| 2OLE | 0.015 | **0.615** | 0.016 | 0.159 | 0.006 |
| 1O3OL | 0.146 | 0.007 | **0.238** | 0.233 | 0.170 |
| Eigenvalue | 5.220 | 3.162 | 1.931 | 1.351 | 1.049 |
| Variability (%) | 34.802 | 21.077 | 12.875 | 9.004 | 6.994 |
| Cumulative % | 34.802 | 55.879 | 68.754 | 77.758 | 84.753 |

(2AP; 2-acetyl-1-pyrroline, PL;pentanal, HL-hexanal, HPL;Heptanal, OL;Octanal, NL;Nonanal, 2NEL; (E)-2-Nonenal, DL;Decanal, PAL; Phenylacetaldehyde, 1ONL; 1-Octanol, 3O2NE; (E)-3-Octen-2-one, 2PF; 2-Pentylfuran, 2OLE; (E)-2-Octenal, 1O3OL; 1-Octen-3-ol)
