# Supplementary figures and images for: Aroma volatile analyses and 2AP characterization at various developmental stages in Basmati and Non-Basmati scented rice (Oryza sativa L.) cultivars
Source: Rice (N Y). 2016 Aug 5;9:38. doi: 10.1186/s12284-016-0113-6 (PMC4975739; doi:10.1186/s12284-016-0113-6)

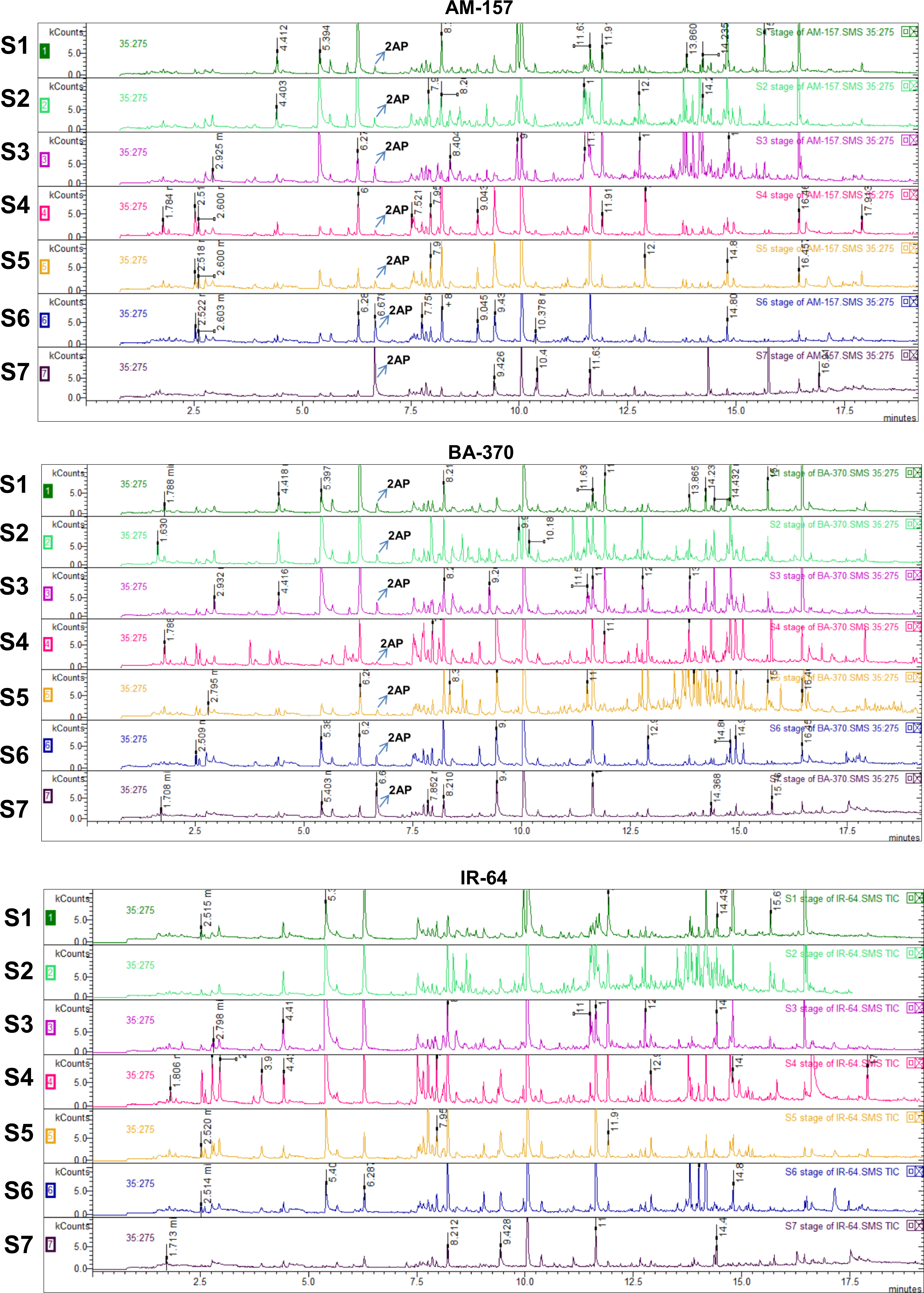

Supplement: Additional file 3: Figure S1. — Comparative GC chromatograms at 7 developmental stages in 3 rice cultivars, S1; seedlings, S2; tillering, S3; booting, S4; flowering, S5; milky grains, S6; dough grains, S7; mature grains. (TIFF 9631 kb) [file 12284_2016_113_MOESM3_ESM.tiff]

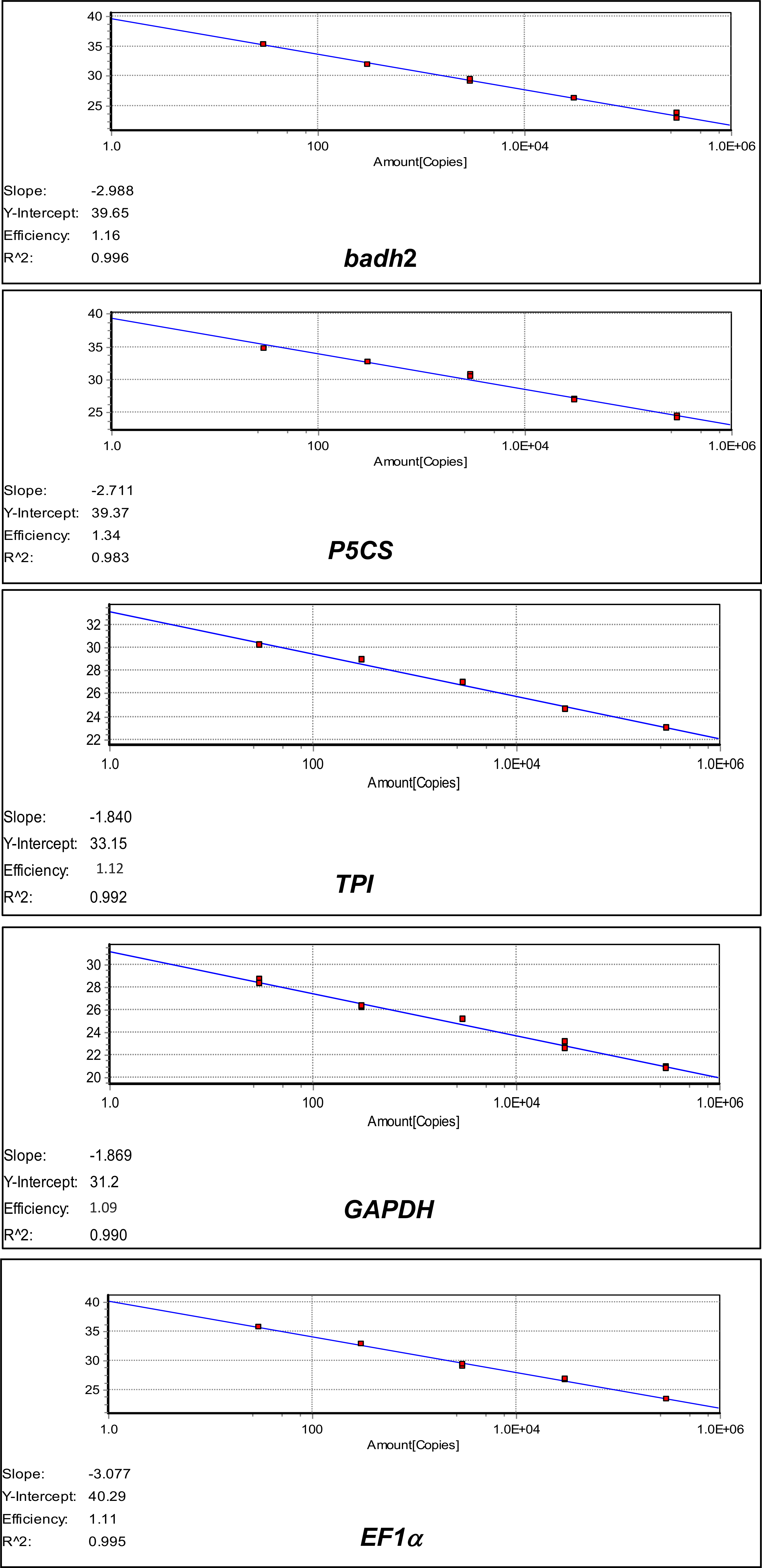

Supplement: Additional file 4: Figure S2. — Standard curves developed for badh2, P5CS, TPI, GAPDH and EF1α genes. (TIFF 1042 kb) [file 12284_2016_113_MOESM4_ESM.tiff]

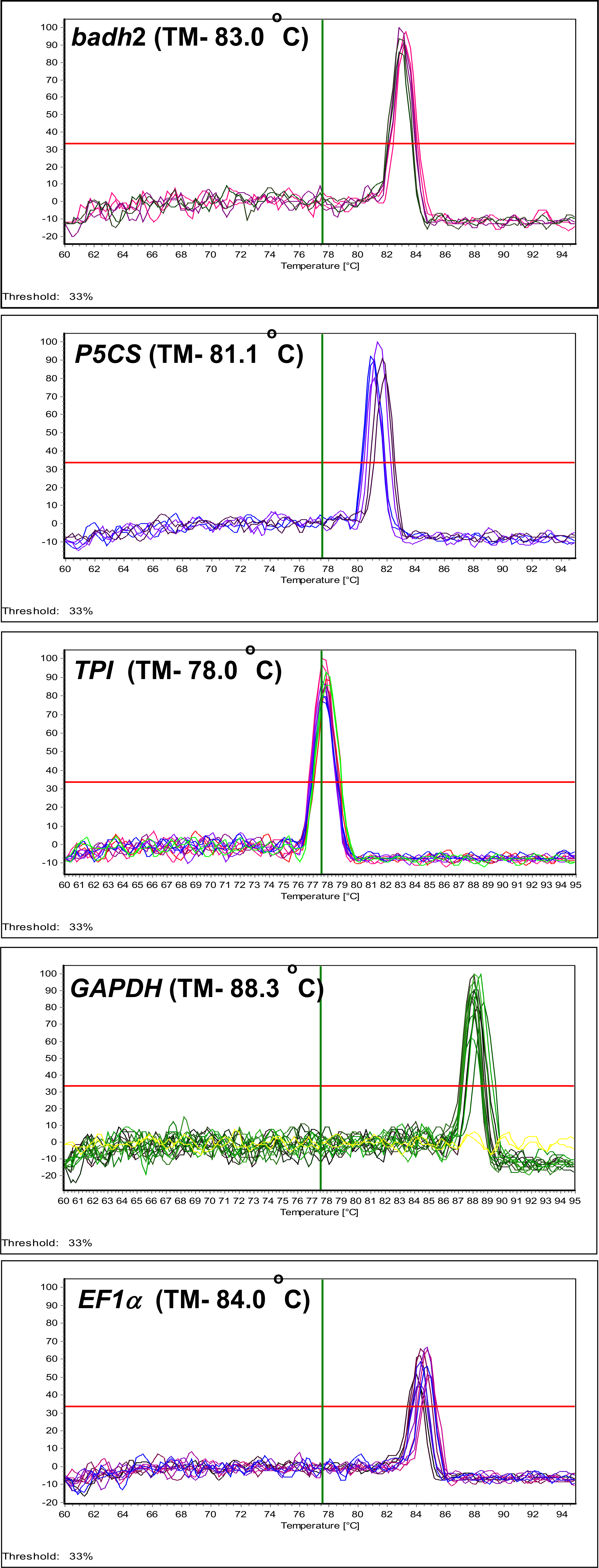

Supplement: Additional file 5: Figure S3. — Melt curves of badh2, P5CS, TPI, GAPDH and EF 1α genes. (TIFF 2645 kb) [file 12284_2016_113_MOESM5_ESM.tiff]
